# Dysregulation of p53-RBM25-mediated circAMOTL1L biogenesis contributes to prostate cancer progression through the circAMOTL1L-miR-193a-5p-Pcdha pathway

**DOI:** 10.1038/s41388-018-0602-8

**Published:** 2018-12-07

**Authors:** Zhan Yang, Chang-Bao Qu, Yong Zhang, Wen-Feng Zhang, Dan-Dan Wang, Chun-Cheng Gao, Long Ma, Jin-Suo Chen, Kai-Long Liu, Bin Zheng, Xin-Hua Zhang, Man-Li Zhang, Xiao-Lu Wang, Jin-Kun Wen, Wei Li

**Affiliations:** 1 Department of Urology, The Second Hospital of Hebei Medical University, 215 Heping W Rd, Shijiazhuang, 050000 China; 2 Department of Biochemistry and Molecular Biology, Ministry of Education of China, Hebei Medical University, No. 361 Zhongshan E Rd, Shijiazhuang, 050017 China; 3 Department of Science and Technology, The Second Hospital of Hebei Medical University, 215 Heping W Rd, Shijiazhuang, 050000 China; 4 Department of Emergency Medicine, The second hospital of Hebei Medical University, Shijiazhuang, Hebei 050000 China

**Keywords:** Metastasis, Focal adhesion, Non-coding RNAs

## Abstract

p53, circRNAs and miRNAs are important components of the regulatory network that activates the EMT program in cancer metastasis. In prostate cancer (PCa), however, it has not been investigated whether and how p53 regulates EMT by circRNAs and miRNAs. Here we show that a Amotl1-derived circRNA, termed circAMOTL1L, is downregulated in human PCa, and that decreased circAMOTL1L facilitates PCa cell migration and invasion through downregulating E-cadherin and upregulating vimentin, thus leading to EMT and PCa progression. Mechanistically, we demonstrate that circAMOTL1L serves as a sponge for binding miR-193a-5p in PCa cells, relieving miR-193a-5p repression of *Pcdha* gene cluster (a subset of the cadherin superfamily members). Accordingly, dysregulation of the circAMOTL1L-miR-193a-5p-Pcdha8 regulatory pathway mediated by circAMOTL1L downregulation contributes to PCa growth in vivo. Further, we show that RBM25 binds directly to circAMOTL1L and induces its biogenesis, whereas p53 regulates EMT via direct activation of *RBM25* gene. These findings have linked p53/RBM25-mediated circAMOTL1L-miR-193a-5p-Pcdha regulatory axis to EMT in metastatic progression of PCa. Targeting this newly identified regulatory axis provides a potential therapeutic strategy for aggressive PCa.

## Introduction

Prostate cancer (PCa) is one of the most common carcinoma among men worldwide. Organ-confined PCa can be effectively treated via radical prostatectomy [1]. However, for advanced PCa, androgen deprivation therapy (ADT) is considered as a first-line treatment [2]. Once hormone resistance develops, advanced PCa is often fatal due to metastasis of PCa [3]. It is well known that PCa metastasis is closely associated with epithelial–mesenchymal transition (EMT) process whereby epithelial cells lose their ability to adhere to adjacent cells and extracellular matrix proteins and acquire mesenchymal phenotype [4]. E-cadherin, a key epithelial marker, enables the cells to maintain epithelial phenotype and is responsible for adherens junction, and β-catenin and vimentin are mesenchymal markers needed for cellular migration [4]. EMT involves the downregulation of E-cadherin and upregulation of β-catenin and vimentin, thus leading to the decrease of intercellular adhesion and increase of cell migration [5]. Clustered protocadherin-α (Pcdha, 14 isoforms) belongs to a subset of the cadherin superfamily, a group of cell adhesion molecules. They share significant sequence homology with classic cadherins in their extracellular domain and mediate both hemophilic and heterophilic cell-cell adhesion [6, 7]. Although some research has revealed that clustered Pcdhs are mainly expressed in nervous system and play critical roles in neurodevelopment [8, 9], a member of this superfamily, PCDHGC3, is also highly expressed in normal colonic epithelium, while its expression is suppressed in carcinoma cell lines [10], and PCDH10 is also downregulated in gastric cancer cell lines [11], suggesting that Pcdhs may act as tumor suppressors through influencing tumor growth and metastasis. Although recent studies have revealed multiple roles for the Pcdhs in the context of tumor biology, we still know relatively little about how the expression of EMT-related proteins, including Pcdha, is regulated in PCa growth and metastasis.

EMT involves drastic changes in gene expression patterns as well as in cell behavior including adhesion, migration, proliferation and apoptosis [5]. The expression of genes involved in EMT is regulated by a complex network of factors that includes cytokines, growth factors, signaling pathways, transcription factors, and the tumor microenvironment [12]. For example, EMT-related transcription factors such as SNAI1/Snail1, SNAI2/Snail2, ZEB1, ZEB2, TCF3 and Kruppel-like factor 8 (KLF8) can bind to the E-cadherin promoter and repress its transcription, whereas factors such as Twist, Goosecoid, TCF4, homeobox protein SIX1 and fork-head box protein C2 (FOXC2) repress E-cadherin indirectly [13, 14]. Besides E-cadherin, these transcription factors also suppress transcriptionally other junctional proteins, including claudins and desmosomes. Moreover, several signaling pathways (TGF-β, FGF, PDGF, EGF, HGF, IGF, ERα, Wnt/beta-catenin and Notch) and hypoxia are involved in EMT [15–17]. These signaling pathways ultimately activate the transcription of EMT-related transcription factors. In addition, recent studies have shown that EMT is also regulated by post-transcriptional mechanisms. For example, the miR-200 family, miR-101and miR-506 have been found to control EMT by regulating EMT-related transcription factors or EMT-related gene expression [15]. miR-27b and miR-34a enhance docetaxel sensitivity of PCa cells through inhibiting EMT by targeting ZEB1 [18]. Recently, we also found that silencing of miR-193a-5p increases the chemosensitivity of PCa cells to docetaxel [19]. These observations strongly suggest that miRNA families are involved in the regulatory network of EMT and promote tumor metastasis by inducing EMT. Despite the involvement of microRNAs in the regulation of EMT, how these microRNAs are regulated in PCa progression remains largely unknown.

Circular RNAs (circRNAs), a class of noncoding RNAs formed by back-splicing of exons, have recently been confirmed to play specific biological roles in EMT. A recent study has revealed that hundreds of circRNAs are induced during human EMT [20]. Kong et al. found that androgen-responsive circSMARCA5, which is induced during EMT, is upregulated and promotes cell proliferation in prostate cancer [21]. Our previous study found that ERβ-suppressed circATP2B1 led to reduced miR-204–3p, which increased fibronectin 1 (FN1) expression and enhanced ccRCC cell invasion [22]. Moreover, a epithelial tight junction-related gene, angiomotin-like 1 gene (Amotl1), has been shown to form circular RNAs (circ-Amotl1) [23, 24], a probable indication for a potential functional role in EMT. CircRNAs are now known to regulate eukaryotic gene expression by acting as cytoplasmic microRNA sponges, RNA-binding protein-sequestering agents, or nuclear transcriptional regulators [25]. It has also been demonstrated that circRNAs result from a non-canonical form of alternative splicing, and that RNA-binding proteins (RBPs) are crucial for the formation of circRNAs [26, 27]. Despite the recent advances in understanding the roles of EMT-related proteins, microRNAs, circRNAs, and RBPs in the pathophysiology of EMT induced by different factors, the intrinsic relationship between these regulatory networks has not been clarified.

Here we show that Amotl1-derived circAMOTL1L is downregulated in human PCa, and that decreased circAMOTL1L facilitates PCa cell migration and invasion through downregulating E-cadherin and increasing vimentin expression, thus leading to EMT and PCa progression. Mechanistically, we demonstrate that circAMOTL1L serves as a sponge for binding miR-193a-5p in PCa cells, relieving miR-193a-5p repression of Pcdha gene cluster (a subset of the cadherin superfamily members). Accordingly, dysregulation of the circAMOTL1L-miR-193a-5p-Pcdha8 regulatory pathway mediated by circAMOTL1L downregulation contributes to PCa growth in vivo. Further, we show that RBM25 binds directly to circAMOTL1L and induces its biogenesis, whereas p53 regulates EMT-related gene expression via RBM25-mediated circAMOTL1L formation. These findings let us to link p53-RBM25-mediated circAMOTL1L-miR-193a-5p-Pcdha regulatory axis to EMT in PCa growth and metastasis, providing a novel insight into regulatory network that induces EMT.

## Results

### CircAMOTL1L is downregulated in human PCa tissues and different PCa cell lines

**Fig. 1:**
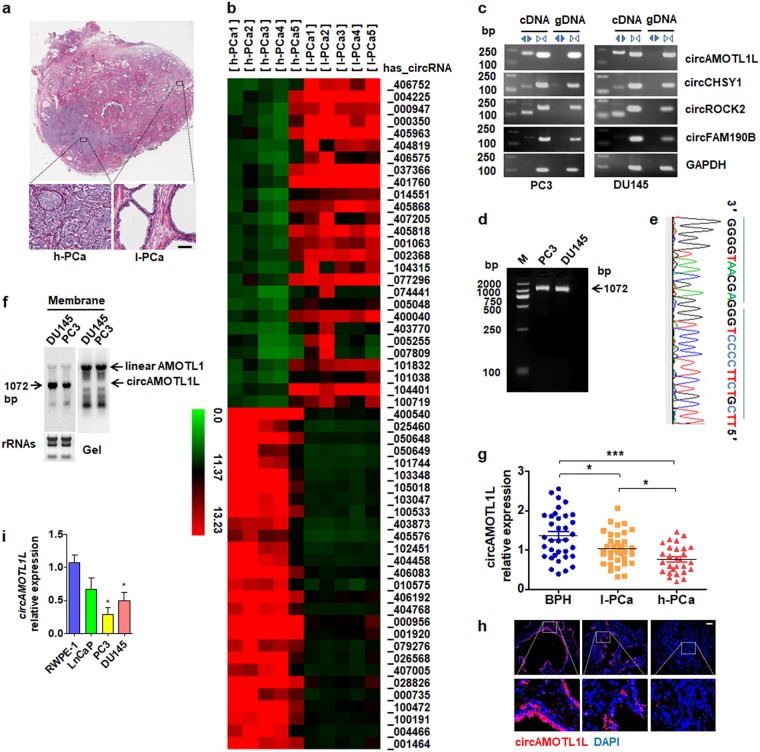
Analysis of circular RNA expression in human PCa tissues and cell lines. **a** High-quality digital slide systems were used to scan a whole cross-section of prostate cancer and demonstrated the heterogeneity in human PCa tissues. The areas of high-grade PCa (Gleason>8; h-PCa) and low-grade PCa (Gleason<6; l-PCa) were enlarged in the prostatic peripheral zone. **b** Differential circRNA expression profiles in high-grade (h-PCa) and low-grade PCa (l-PCa) tissues. Heat map of hierarchical clustering indicates differentially expressed circRNAs (red: upregulation; green: downregulation). A number in the right side represents a circular RNA, such as _406752 represents has_circRNA_406752. **c** Convergent or divergent primers were used to detect the indicated circRNAs via reverse transcription (RT)-PCR in PC3 and DU145 PCa cell lines. circRNAs were amplified by divergent primers in cDNA but not genomic DNA (gDNA) and linear control gene GAPDH. bp: size markers (in base pars). **d** RT-PCR amplified full-length has_circRNA_000350 (circAMOTL1L) in PC3 and DU145 cell lines and amplified products were confirmed by agarose gel electrophoresis. **e** Sanger sequencing confirmed head-to-tail splicing of circAMOTL1L. **f** Northern blotting detected circAMOTL1L and linear AMOTL1 in PC3 and DU145 cell lines. **g** Quantitative real-time (qRT)-PCR analysis detected circAMOTL1L expression in benign prostatic hyperplasia (BPH, *n* = 35), low-grade PCa (l-PCa, *n* = 34) and high-grade PCa (h-PCa, *n* = 28) using divergent primers. *P < 0.05 vs. BPH or l-PCa, ***P < 0.001 vs BPH. **h** Fluorescence In situ hybridization (FISH) of circAMOTL1L (red) combined with nuclear DAPI staining (blue) in BPH, low-grade PCa and high-grade PCa tissues. Scale bar=32 μm. **i** Expression of circAMOTL1L in non-cancer and cancer cell lines from the prostate tissues was analyzed by qRT-PCR. Bars are mean ± SEM of triplicate samples. *P < 0.05 vs. RWPE-1 cells

To explore the molecular mechanisms underlying circRNA role in prostate carcinogenesis and progression of PCa, we first used circular RNA microarrays to acquire circRNA profiles in PCa tissues. Five pairs of samples were taken from the two different areas, i.e., high-grade PCa (Gleason>8) and low-grade PCa (Gleason< 6), within the same PCa sample, as shown in Fig. 1a. After scanning and normalization, 2238 circRNAs were found to be differentially expressed between high-grade PCa tissues and low-grade PCa tissues (>2.0 fold change in expression level; P <0.05). Among them, 1238 circRNAs were upregulated and 1000 were downregulated in high-grade PCa tissues (Supplementary Fig. 1 and Supplementary Table 1). As shown in Fig. 1b, unsupervised hierarchical clustering analysis of circRNA profiling was able to clearly differentiate the high-grade PCa from the low-grade PCa. To further confirm the microarray data, four differentially expressed circRNAs were selected to verify their expressions in PC3 and DU145 cell lines by using qRT-PCR. We used divergent primers to amplify circRNAs formed by head-to-tail splicing and confirmed the presence of those circRNAs, which were detected by the microarray screening, in PC3 and DU145 cell lines (Fig. 1c). Next, we designed additional divergent primers with partially overlapping 5′-end nucleotide bases to identify full-length circRNAs. RT-PCR and Sanger sequencing revealed that one stably expressed circRNA, termed circAMOTL1L (hsa_circRNA_000350), was amplified (Fig. 1d and e). Using Northern blotting, we analyzed circAMOTL1L and its corresponding linear RNA and demonstrated that circAMOTL1L was derived from circularization of the exon-2 and exon-3 of the AMOTL1 gene (Fig. 1f). Bioinformatics analysis revealed that the exon-2 upstream intron and the exon-3 downstream intron in the *AMOTL1* gene contain, respectively, a long flanking sequence with complementary Alu repeats, which might facilitate the cyclization of a circRNA (Supplementary Fig. 2) [28, 29].

To provide additional confirmation that circAMOTL1L is differentially expressed in human PCa, prostatectomy specimens of patients with high-grade PCa (Gleason>8), low-grade PCa (Gleason<6) and benign prostatic hyperplasia (BPH) were examined by qRT-PCR using divergent primers. Consistent with the microarray results, circAMOTL1L expression was significantly downregulated in high-grade PCa tissues compared with low-grade PCa or BPH tissues. Moreover, the circAMOTL1L expression level has a significant association with Gleason score (P < 0.05) and preoperative PSA (P < 0.05), but not with patient’s age. As expected, expression level of circAMOTL1L was obviously lower in low-grade PCa tissues than in BPH tissues (Fig. 1g). In parallel, the same results were obtained by fluorescence in situ hybridization (FISH) using a probe against circAMOTL1L (Fig. 1h). These results suggest that the downregulation of circAMOTL1L might be related to prostate carcinogenesis. In further experiments, we evaluated circAMOTL1L expression in cancerous and non-cancerous prostate cell lines and observed that the expression level of circAMOTL1L was significantly reduced in PCa cell lines PC3 and DU145, particularly in PC3 cells, compared to normal prostate epithelial cells (RWPE-1) (Fig. 1i). Taken together, our results suggest that circAMOTL1L expression is downregulated in PCa tissues and different PCa cell lines.

### CircAMOTL1L regulates PCa cell migration and invasion by modulating the expression of EMT-related genes in vitro

**Fig. 2:**
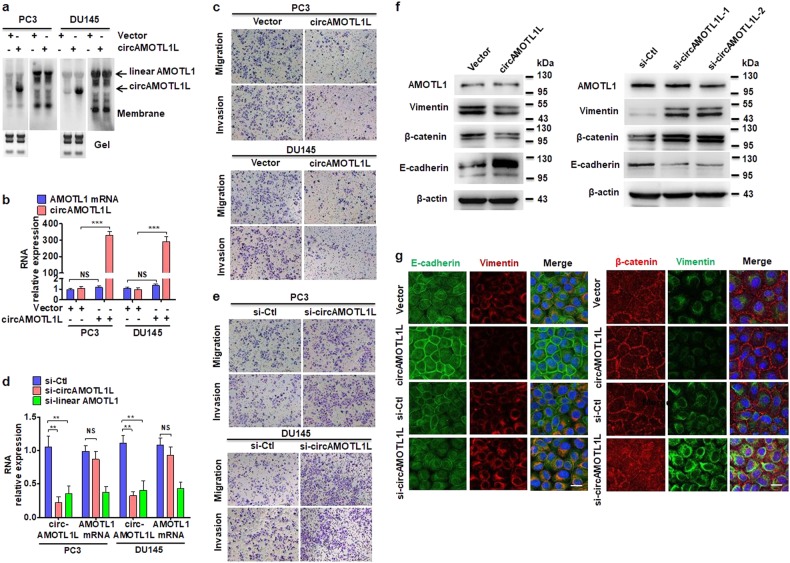
circAMOTL1L regulates PCa cell migration and invasion by modulating the expression of EMT-related genes in vitro. **a** Northern blot analysis detected circAMOTL1L and linear AMOTL1 in PC3 and DU145 cells stably transfected with circAMOTL1L (pcDNA-circAMOTL1L) or empty vector (circ-pcDNA3.1). **b** qRT-PCR examined the expression of circAMOTL1L and AMOTL1 mRNA in PC3 and DU145 cells treated as in (a). ***P<0.001 vs. empty vector. **c** Cell migration and invasion of PC3 and DU145 cells treatment as above were evaluated by transwell assay. **d** qRT-PCR detected circAMOTL1L and AMOTL1 mRNA expression in PC3 and DU145 cells transfected with si-circAMOTL1L, si-linear AMOTL1 or si-Ctl. Bars are mean ± SEM of triplicate samples. **P < 0.05 vs. si-Ctl. **e** Cell migration and invasion of PC3 and DU145 cells transfected with indicated siRNAs were detected by transwell assay. **f** PC3 cells were transfected with circAMOTL1L or empty vector (left) as well as with si-Ctl or two siRNAs targeting different regions of AMOTL1 gene (right) for 24 h, and then EMT-related proteins vimentin, β-catenin, E-cadherin, and AMOTL1 were determined by western blot analysis. β-actin was used as an internal control. **g** Immunofluorescence staining was used to examine expression of E-cadherin, vimentin and β-catenin in PC3 cells with indicated antibodies. Scale bars=20 μm. All experiments were performed in triplicate

To investigate the function of circAMOTL1L in PCa cells, we constructed a recombinant plasmid expressing circRNAs (pcDNA-circAMOTL1L) that could completely and non-redundantly express circAMOTL1L. The integrity and overexpression of circAMOTL1L were confirmed in pcDNA-circAMOTL1L-transfected PC3 and DU145 cells by Northern blotting (Fig. 2a). Overexpression of circAMOTL1L significantly increased circAMOTL1L but did not affect linear mRNA level in both tested cell lines (Fig. 2a and b). To assess the role of circAMOTL1L in PCa pathophysiology, PC3 and DU145 cells were transfected with pcDNA-circAMOTL1L and allowed to migrate or invade, as determined by a transwell membrane, wound healing or matrigel invasion assays. As shown in Fig. 2c and Supplementary Fig. 3a, there was a significant decrease in the number of circAMOTL1L-overexpressing PC3 and DU145 cells that migrated or invaded compared to the empty vector-transfected cells. Consistently, a scratch wound healing assay also showed that circAMOTL1L overexpression significantly inhibited the migration of PC3 and DU145 cells (Supplementary Fig. 3b and c). Because circRNAs can be silenced by short interfering RNA (siRNA) [28], we designed two siRNAs: one siRNA targeting the head-to-tail splicing sequence (si-circAMOTL1L), and another siRNA targeting sequence in a circularized exon shared by both linear and circular RNA sequence (si-linear AMOTL1) to knock down the expression of circAMOTL1L, or circAMOTL1L together with AMOTL1 mRNA. As expected, si-circAMOTL1L only reduced the circAMOTL1L transcript but did not affect linear AMOTL1 RNA, whereas si-linear AMOTL1 effectively knocked down both transcripts (Fig. 2d), indicating that siRNA-mediated circAMOTL1L knockdown is very specific. Further, we examined the effect of circAMOTL1L knockdown on migration and invasion of PCa cell lines. The results showed that circAMOTL1L knockdown in PC3 and DU145 cells dramatically increased cell migration and invasion, as shown by a transwell membrane or matrigel invasion assays (Fig. 2e and Supplementary Fig. 3d). Moreover, the similar results were obtained by wound healing assays, showing that knockdown of circAMOTL1L significantly promoted PC3 and DU145 cell migration (Supplementary Fig. 3e and f). These data clearly suggested that circAMOTL1L plays important roles in prostate carcinogenesis and tumor progression through regulating migration and invasion of PCa cells.

Because epithelial–mesenchymal transition (EMT) enables stationary epithelial cells to gain the ability to migrate and invade adjacent tissues [30], we sought to know whether circAMOTL1L affects migration and invasion of PCa cells via regulating the expression of EMT-related proteins. Thus, expression of EMT-related proteins, E-cadherin, vimentin and β-catenin were examined in circAMOTL1L-overexpressed or depleted PC3 cells. The results showed that overexpression of circAMOTL1L dramatically increased expression of the epithelial marker E-cadherin and reduced expression of the mesenchymal markers, vimentin and β-catenin, but did not affect AMOTL1 protein level, in PC3 cells. Conversely, depletion of circAMOTL1L in PC3 cells markedly decreased E-cadherin and elevated vimentin and β-catenin protein levels (Fig. 2f).

Immunofluorescence staining of circAMOTL1L-overexpressed or depleted PC3 cells for E-cadherin, vimentin and β-catenin showed that circAMOTL1L overexpression markedly promoted E-cadherin expression, with an obvious increase in membrane localization of E-cadherin and β-catenin, but obviously reduced vimentin expression. However, knockdown of circAMOTL1L led to a loss of E-cadherin in the cell membrane and an increased vimentin and β-catenin expression, with a significant increase in nuclear localization of β-catenin (Fig. 2g). Together, our findings demonstrate that circAMOTL1L contributes to EMT via regulation of E-Cadherin, vimentin and β-catenin in PCa cell lines. Combined with the previous studies demonstrating that β-catenin regulates the expression of genes involved in cell migration and adhesion, such as cadherins and catenins [31], our data imply that increased nuclear localization of β-catenin in circAMOTL1L-depleted PC3 cells could further aggravate the dysregulation of EMT-related protein expression.

### CircAMOTL1L serves as a sponge for binding miR-193a-5p in PCa cells

**Fig. 3:**
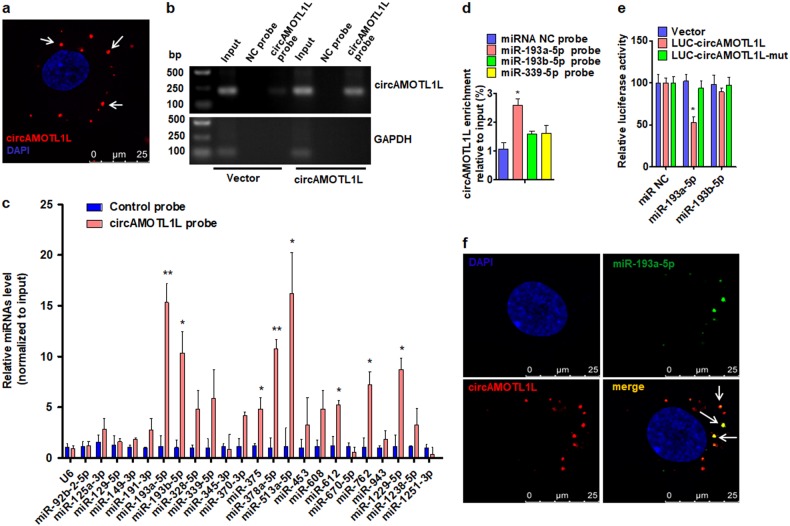
circAMOTL1L serves as a sponge for miR-193a-5p in PCa cells. **a** RNA fluorescence in situ hybridization for the detection of circAMOTL1L (*n* = 13), showing the localization of circAMOTL1L (detected with a junction probe). The cell nuclei were counterstained blue by 4′,6-diamidino-2-phenylindole (DAPI). Scale bar=25 μm. **b**, PC3 cells were transfected with circAMOTL1L (pcDNA-circAMOTL1L) or empty vector, and then RT-PCR was used to detect circAMOTL1L in the precipitates pulled down with biotin-labeled probe against circAMOTL1L. GAPDH was used as negative control. **c**, The microRNAs were pulled down from PC3 cell lysates with biotin-labeled circAMOTL1L or NC probe, and qRT-PCR was used to detect the relative level of indicated microRNAs. Graph bars are mean ± SEM of 3 independent experiments. *P<0.05, **P<0.01 vs. NC probe. **d** circAMOTL1L was sedimented by biotin-labeled miR-193a-5p, miR-193b-5p, miR-339–5p, or NC probe, and qRT-PCR was used to determine circAMOTL1L enrichment. Graph bars are mean ± SEM of 3 independent experiments. *P<0.05 vs. NC probe. **e** The luciferase activity of LUC-circAMOTL1L or LUC-circAMOTL1L-mut reporter constructs was measured in PC3 cells co-transfected with miR-193a-5p mimic, miR-193b-5p mimic or NC miR. Graph bars are mean ± SEM of 3 independent experiments. *P<0.05 vs. empty vector. **f** RNA in situ hybridization detected the co-localization between miR-193a-5p and circAMOTL1L (arrowheads) in PC3 cells co-transfected with expression vectors for circAMOTL1L and miR-193a-5p. Nuclei were counterstained with DAPI. Scale bars = 25 μm

Because circRNAs can function as miRNA sponges in the cytoplasm or regulate gene expression in the nucleus as nuclear transcriptional regulators, we first performed a FISH with a probe against circAMOTL1L to pinpoint the localization of circAMOTL1L. As shown in Fig. 3a, circAMOTL1L was mainly localized in the cytoplasm. We next searched for microRNA-binding sites in the circAMOTL1L sequence and their corresponding heteroduplexes via bioinformatics analysis [32]. The results showed that circAMOTL1L contains sequences complementary to miR-193a-5p, miR-193b-5p, miR-125a-5p, miR-339–5p and miR-92a-2–5p. Among these miRNAs, circAMOTL1L harbors 6 putative binding sites for miR-193a-5p (Supplementary Fig. 4). Subsequently, we performed a pull-down assay using a biotin-labeled circAMOTL1L probe in PCa cells and found that the pull-down efficiency of circAMOTL1L was markedly enhanced in circAMOTL1L-overexpressed cells (Fig. 3b). In further experiments, the level of the candidate miRNAs in the precipitates pulled down with biotin-labeled circAMOTL1L was detected by real-time PCR. As shown in Fig. 3c, besides the miR-193a-5p and miR-193b-5p, the miR-375, miR-378a-5p, miR-513a-5p, miR-612, miR-762 and miR-1229–5p were also significantly enriched in the precipitates. Additionally, biotin-labeled miR-193a-5p, miR-193b-5p or miR-339–5p were used to pull-down circAMOTL1L, and the qRT-PCR analysis for circAMOTL1L enrichment showed that circAMOTL1L level was significantly elevated in the complexes sedimented by miR-193a-5p, but not miR-193b-5p, miR-339–5p or the negative control miRNA (miRNA NC) (Fig. 3d). Furthermore, we constructed a luciferase reporter plasmid through inserting wild-type circAMOTL1L sequence or its corresponding mutants lacking miR-193a-5p- and miR-193b-5p-binding site into the immediately downstream of the luciferase reporter gene. Luciferase assay revealed that co-transfection with miR-193a-5p, but not miR-193b-5p, significantly decreased luciferase activity mediated by wild-type circAMOTL1L sequence, whereas both miR-193a-5p and miR-193b-5p did not affect luciferase activity mediated by the miRNA binding site-mutated circAMOTL1L (Fig. 3e). Next, RNA in situ hybridization for the detection of miR-193a-5p co-localization with circAMOTL1L was carried out, and the results were consistent with the presence of circAMOTL1L interaction with miR-193a-5p as shown by pull-down assays (Fig. 3f). These findings indicate that circAMOTL1L functions as a sponge of miR-193a-5p.

### miR-193a-5p targets protocadherin-α (Pcdha) gene cluster in PCa cells

**Fig. 4:**
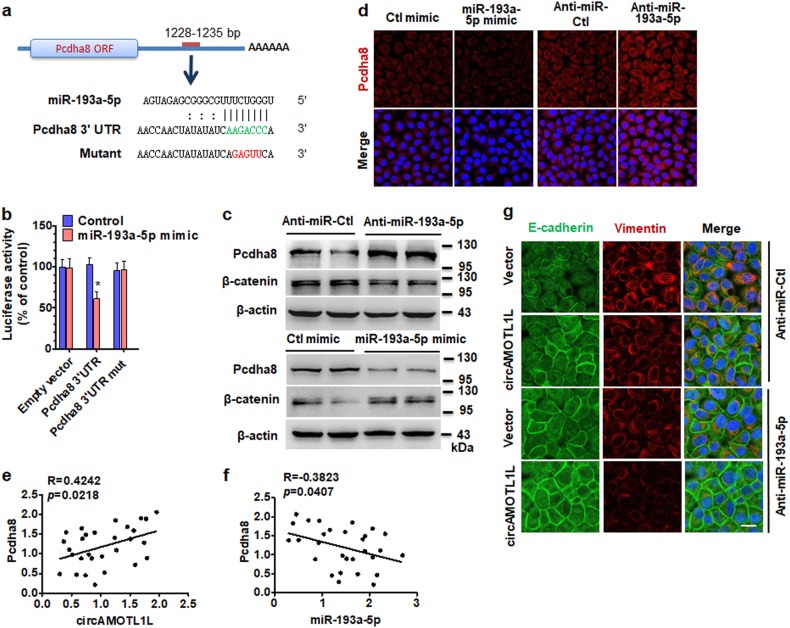
Pcdha8 is a direct target of miR-193a-5p in PCa cells. **a** Potential binding site of miR-193a-5p at the 3′ UTR of human Pcdha8 mRNA is shown in the green color. Red color indicates the sequence of the mutated miR-193a-5p-binding site. **b** PC3 cells were co-transfected with miR-193a-5p mimic or control mimic and pmir-GLO vector containing wild-type or mutated miR-193a-5p-binding site (mut) at Pcdha8 3′-UTR. Luciferase reporter assays were performed. Graph bars are mean ± SEM of 3 independent experiments. *P < 0.05 vs. control. **c** PC3 cells were transfected with anti-miR-193a-5p or miR-193a-5p mimic and their corresponding controls, Pcdha8 and β-catenin expression was analyzed by western blotting. **d** Immunofluorescence staining examined Pcdha8 expression in PC3 cells treated as in (**c**). **e** Pcdha8 mRNA and circAMOTL1L were measured by qRT-PCR, and the Pearson correlation analysis shows a positive correlation between Pcdha8 mRNA and circAMOTL1L (*R* = 0.4242; *P* = 0.0218). **f** Pearson correlation analysis was used to analyze the relationships between miR-193a-5p and Pcdha8 mRNA (*R* = −0.3823, *P* = 0.0407). **g** PC3 cells were co-transfected with circAMOTL1L and anti-miR-193a-5p or their corresponding controls, immunofluorescence staining was used to detect the expression of EMT-related proteins E-cadherin (green) and vimentin (red)

We then examined whether circAMOTL1L-associated miR-193a-5p regulates the expression of EMT-related genes by targeting their 3′ UTR. To do this, we first used miRNA target prediction algorithms, including TargetScan (http://www.targetscan.org) and RNAhybrid [33, 34], to identify target genes of miR-193a-5p. The results showed that among the target genes predicted by bioinformatics analysis, Pcdha gene cluster, a subset of the cadherin superfamily members, contains highly conserved miR-193a-5p-binding site in their 3' UTR (Fig. 4a and Supplementary Fig. 5a). These findings prompted us to investigate whether miR-193a-5p regulates the expression of Pcdha gene cluster. To do this, we transfected PC3 cells with anti-miR-193a-5p to down-regulate miR-193a-5p expression. The qRT-PCR analysis revealed that miR-193a-5p knockdown led to a significant increase in the expression of most genes of Pcdha gene cluster, including Pcdha2, Pcdha3, Pcdha4, Pcdha5, Pcdha7, Pcdha8, Pcdha9, and Pcdha11. Among the Pcdha genes examined, Pcdha8 was found to be the most significant upregulated (Supplementary Fig. 5b). Because the Pcdha gene cluster shares the same 3' UTR sequence, one of these genes, Pcdha8, was chosen to conduct all subsequent experiments. We cloned the Pcdha8 3' UTR sequence containing the miR-193a-5p-binding site or its mutant into luciferase reporter plasmid and co-transfected PC3 cells with these constructs and miR-193a-5p mimic. As expected, miR-193a-5p mimic significantly decreased the luciferase activity mediated by wild-type Pcdha8 3' UTR but had no effect on the luciferase activity mediated by its mutant (Fig. 4b).

To obtain more evidence supporting the role of miR-193a-5p, we transfected PC3 cells with anti-miR-193a-5p, miR-193a-5p mimic or their corresponding control RNA and detected Pcdha8 protein expression. As shown in Fig. 4c, depletion of miR-193a-5p by its antagomir obviously increased Pcdha8 expression level and reduced expression of β-catenin protein, consistent with the results of circAMOTL1L overexpression. Conversely, miR-193a-5p mimic markedly decreased Pcdha8 protein expression and enhanced β-catenin level. Similarly, immunofluorescence staining of miR-193a-5p mimic- or anti-miR-193a-5p-transfected PC3 cells for Pcdha8 showed that miR-193a-5p overexpression decreased and knockdown of miR-193a-5p increased Pcdha8 expression (Fig. 4d). In further experiments, we examined Pcdha8 expression in PCa and BPH tissues by using qRT-PCR and found that Pcdha8 mRNA levels were significantly lower in PCa tissues than in BPH (Supplementary Fig. 6). Correlation analysis between Pcdha8 mRNA and circAMOTL1L reveals a significant positive correlation between the two RNA levels (Fig. 4e). Previous studies by our lab have demonstrated that miR-193a-5p level was significantly upregulated in PCa cells [19]. We found here that the expression level of miR-193a-5p was negatively correlated with the expression of Pcdha8 in PCa tissues (Fig. 4f). These findings indicate that circAMOTL1L regulates the expression of Pcdha gene cluster through acting as a sponge for binding miR-193a-5p in PCa cells. Immunofluorescence staining also revealed that knockdown of miR-193a-5p by its antagomir increased E-cadherin expression in the cell membrane and reduced vimentin level. Moreover, depletion of miR-193a-5p combined with circAMOTL1L overexpression further enhanced E-cadherin expression and decreased vimentin level (Fig. 4g). Collectively, these results provide strong evidence supporting the notion that miR-193a-5p negatively regulates the expression of Pcdha gene cluster in PCa cells.

### CircAMOTL1L-miR-193a-5p-Pcdha8 pathway is involved in prostate tumorigenesis in vivo

**Fig. 5:**
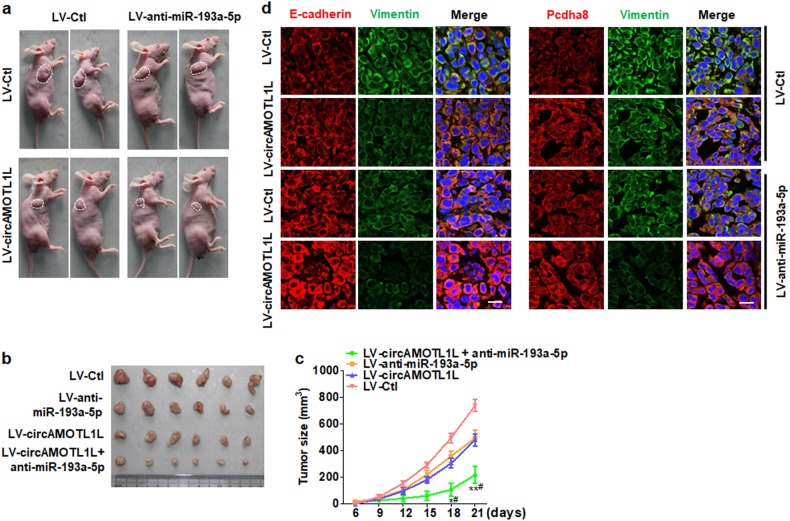
Dysregulation of circAMOTL1L/miR-193a-5p/Pcdha8 axis promotes the growth of PCa in vivo. **a**, **b** PC3 cells, engineered to stably overexpress circAMOTL1L (LV-circAMOTL1L), anti-miR-193a-5p (LV-anti-miR-193a-5p) or both (LV-Ctl serves as the negative control), were injected subcutaneously in 200 μl PBS/Matrigel (50 : 50) into the right forelimb of nude mice to establish xenograft tumors. At the final time point (21 days after injection), the tumor volumes in each group were measured both in situ (**a**) and after resection of tumors (**b**) (*n* = 15 in each group). **c** Tumor volume was determined by direct measurement with calipers and calculated by the formula: volume = [(length × width^2^) / 2]. *P < 0.05, **P < 0.01 vs. LV-anti-miR-193a-5p; #*P* < 0.05 vs. LV-circAMOTL1L (*n* = 15 in each group). **d** Immunofluorescence staining was performed to detect the expression of E-cadherin, vimentin and Pcdha8 in xenograft tumor tissues in vivo. Scale bars = 20 μm

To determine pathophysiological role of the circAMOTL1L-miR-193a-5p-Pcdha8 pathway in prostate tumorigenesis, we established PCa xenograft models by implanting PC3 cells stably overexpressing circAMOTL1L, anti-miR-193a-5p (miR-193a-5p inhibitor), or both into nude mice. As expected, the tumor volumes were significantly decreased in nude mice implanted with circAMOTL1L-overexpressed PC3 cells or with miR-193a-5p-depleted PC3 cells compared with their corresponding control. circAMOTL1L overexpression combined with miR-193a-5p depletion in PC3 cells further reduced tumor growth (Fig. 5a and b). Moreover, the mean tumor volumes in nude mice implanted with PC3 cells infected with LV-circAMOTL1L plus LV-anti-miR-193a-5p were significantly decreased compared to each of them alone (Fig. 5c). Further, we detected the expression of E-cadherin, Pcdha8 and vimentin genes in the xenograft tumor tissues by immunofluorescent staining. As shown in Fig. 5d, overexpression of circAMOTL1L or miR-193a-5p depletion apparently increased E-cadherin and Pcdha8 expression but reduced vimentin protein level. Moreover, the expression changes in these genes were further enhanced when circAMOTL1L overexpression was combined with miR-193a-5p depletion. The results obtained with the recovered tumor materials supported the in vitro data (Supplementary Fig. 7). These data clearly suggest that dysregulation of the circAMOTL1L-miR-193a-5p-Pcdha8 regulatory pathway mediated by circAMOTL1L downregulation contributes to the tumorigenesis of PCa in vivo.

### RBM25 mediates p53 regulation of circAMOTL1L biogenesis

**Fig. 6:**
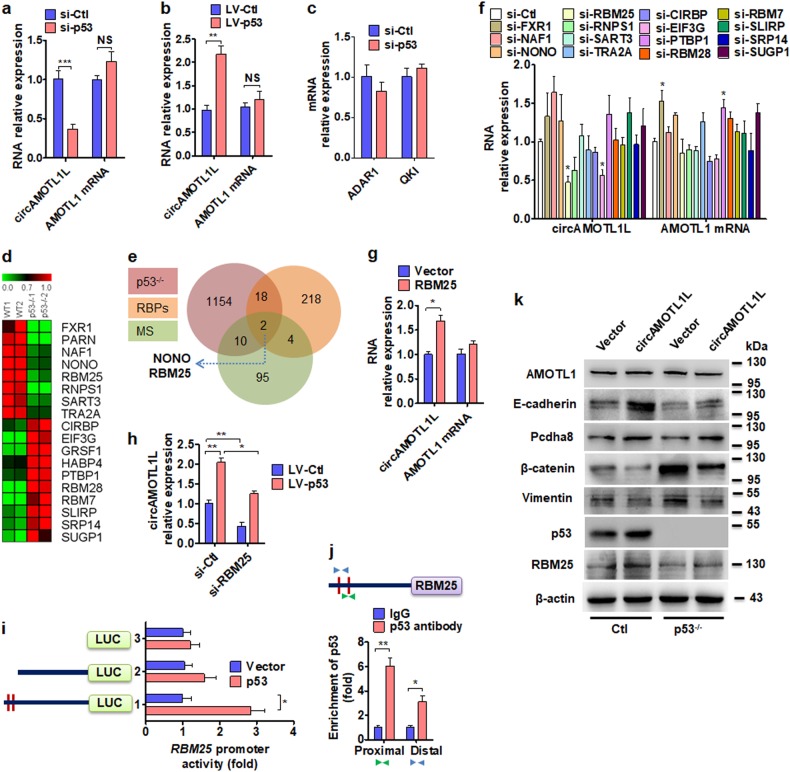
RBM25 mediates p53-regulated circAMOTL1L biogenesis. **a**, **c** PC3 cells were transfected with p53-specific siRNA (si-p53) or control siRNA (si-Ctl), qRT-PCR was used to determine the expression of circAMOTL1L and AMOTL1 mRNA (**a**) or RNA-editing enzyme ADAR1 and RNA-binding protein Quaking (QKI) mRNA (**c**). ***P < 0.001 vs. si-Ctl. **b** qRT-PCR detected circAMOTL1L and AMOTL1 mRNA in PC3 cells engineered to stably overexpress p53 with a lentivirus (LV)-p53 or negative control LV-Ctl. **P<0.01 vs. LV-Ctl. **d** Microarray analysis. Heat map showing the differential expression (fold changes) of RBP genes between wild-type p53 (WT) and p53 knockout (p53^−/−^) PC3 cells from RNA-seq analysis of two independent samples (biological replicates). Green color indicates genes that are known to be transcriptionally upregulated by p53 (Supplementary Table 3). **e** A Venn diagram displays that NONO and RBM25 are overlapped between RBPs, which were differentially expressed in p53^−/−^ PC3 cells, and circAMOTL1L-binding proteins identified by MS among 218 known RBPs (Uniprot datebase). **f** The 15 selected RBPs were knocked down in PC3 cells by their specific siRNAs, qRT-PCR was used to detect the expression of circAMOTL1L and AMOTL1 mRNA. *P<0.05 vs. si-Ctl. **g** Expression of circAMOTL1L and AMOTL1 mRNA was determined by qRT-PCR in PC3 cells stably transfected with lentivirus (LV)-RBM25 or empty vector. *P<0.05 vs. empty vector. **h** qRT-PCR detected the expression of circAMOTL1L in PC3 cells transfected with LV-p53 alone or together with RBM25-specific siRNA (si-RBM25), LV-Ctl and si-Ctl serve as their respective controls. *P < 0.05 vs. LV-p53+si-Ctl, **P < 0.01 vs. si-Ctl+LV-Ctl. **i** Different truncated RBM promoter-luciferase reporter were co-transfected with p53 expression vector into 293A cells, and luciferase reporter assays were performed. *P < 0.05 vs. empty vector. **j** ChIP-qPCR detected p53 binding to the RBM25 promoter region in PC3 cells. Arrowheads indicate the position of primers used for ChIP- PCR. *P < 0.05, **P < 0.01 vs. IgG. **k** p53-depleted PC3 cells were transfected with circAMOTL1L expression vector or empty vector, Western blot analysis was used to analyze the proteins as indicated. All graph bars are mean ± SEM of 3 independent experiments

We next investigated the underlying mechanism of circAMOTL1L decrease in PCa cells. Because p53 gene is frequently mutated or its expression is downregulated in PCa cells [35, 36], as well as the downregulation in our clinical samples (Supplementary Fig. 8a). Therefore, we knocked down p53 expression with siRNA and detected circAMOTL1L in PC3 cells. Interestingly, knockdown of p53 by si-p53 significantly reduced circAMOTL1L expression by 40% of the negative control siRNA (si-Ctl), but did not affect AMOTL1 mRNA level (Fig. 6a; Supplementary Fig. 8b). Reversely, overexpression of p53 mediated by lentiviral vector (LV-p53) significantly increased circAMOTL1L, but not AMOTL1 mRNA, expression levels in PC3 cells (Fig. 6b; Supplementary Fig. 8c). As RNA-binding proteins (RBPs) are necessary for circRNA biogenesis [20, 37], we then examined whether RBPs QKI and ADAR1, which regulate the biogenesis of a number of circRNAs [20, 37, 38], are responsible for circAMOTL1L biogenesis. The results showed that QKI and ADAR1 were neither regulated by p53 nor affected circAMOTL1L biogenesis in PC3 cells, as shown by knocking down p53, QKI or ADAR1, respectively (Fig. 6c; Supplementary Fig. 8d).

To clarify the effect of p53 on *RBP* gene expression, we knocked out p53 gene in PC3 cells to generate p53 knockout stable cell line (p53^-/-^ PC3 cells) and examined the expression of the known RBP genes by RNA sequencing. As shown in Fig. 6d and Supplementary table 3, a total of 18 RBPs were differentially expressed between the p53^-/-^ PC3 cells and wild-type PC3 cells (8 RBPs downregulated; 10 upregulated). Meanwhile, we used biotinylated circAMOTL1L pull down to capture proteins interacting with circAMOTL1L. Mass spectrometric analysis of the co-precipitated proteins showed that proteins (FDR < 1%) interacted with circAMOTL1L (Supplementary table 4). Importantly, between the differentially expressed RBPs in p53^−/−^ PC3 cells and the RBPs precipitated by circAMOTL1L, two RBPs (NONO and RBM25) were merged among the known 218 RBPs (Supplementary table 5). The venn diagram revealed the intersection (Fig. 6e). Subsequently, we knocked down 15 RBPs, including NONO and RBM25, by using siRNA and examined the expression of circAMOTL1L by qRT-PCR. As shown in Fig. 6f, circAMOTL1L was significantly downregulated in RBM25- or EIF3G-knocked down PC3 cells. Because RBM25 is the only one that not only is regulated by p53 and but also affects circAMOTL1L biogenesis among the known RBPs, we then investigated the role of RBM25 in circAMOTL1L biogenesis. The results showed that RBM25 overexpression significantly increased circAMOTL1L expression but did not affect AMOTL1 mRNA level (Fig. 6g). In further experiments, we overexpressed p53 by using a lentiviral vector system (LV-p53) and knocked down RBM25 expression in PC3 cells with three different siRNAs targeting RBM25. As shown in Fig. 6h and Supplementary Fig. 8e, overexpression of p53 alone increased circAMOTL1L expression 2.0-fold over that seen with the empty vector transfection (LV-Ctl), whereas p53 overexpression together with RBM25 knockdown abolished the inducing effect of p53 upregulation on circAMOTL1L expression. Collectively, these data strongly suggest that RBM25 mediates p53 regulation of circAMOTL1L expression.

To further determine whether RBM25 is a direct transcriptional target of p53, progressive 5’ deletion constructs of the RBM25 promoter fused to the luciferase reporter gene were cloned and transiently transfected into PC3 cells. Luciferase assay revealed that the distal 5 kb promoter region (−3798 to −3783 bp upstream of the transcription start site) is essential for p53-mediated transcription of RBM25 gene (Fig. 6i; Supplementary Fig. 8f). Furthermore, ChIP analysis confirmed that p53 bound predominantly to the distal region of the RBM25 promoter in PC3 cells (Fig. 6j; Supplementary Fig. 8g), suggesting that RBM25 is a direct target of p53. To investigate whether p53 plays a role in circAMOTL1L-regulated EMT, we performed a rescue experiment by overexpressing circAMOTL1L in the p53-depleted PC3 cells. Western blot analysis revealed that depletion of p53 in PC3 cells markedly decreased the expression of E-cadherin, Pcdha8 and RBM25, but not AMOTL1, and increased vimentin and β-catenin expression. However, the downregulation of E-cadherin, Pcdha8 and RBM25 induced by depletion of p53 was partly rescued by overexpression of circAMOTL1L (Fig. 6k). These findings clearly suggest that p53 regulates EMT-related gene expression via RBM25-mediated circAMOTL1L formation.

### RBM25 binds directly to circAMOTL1L and induces its biogenesis

**Fig. 7:**
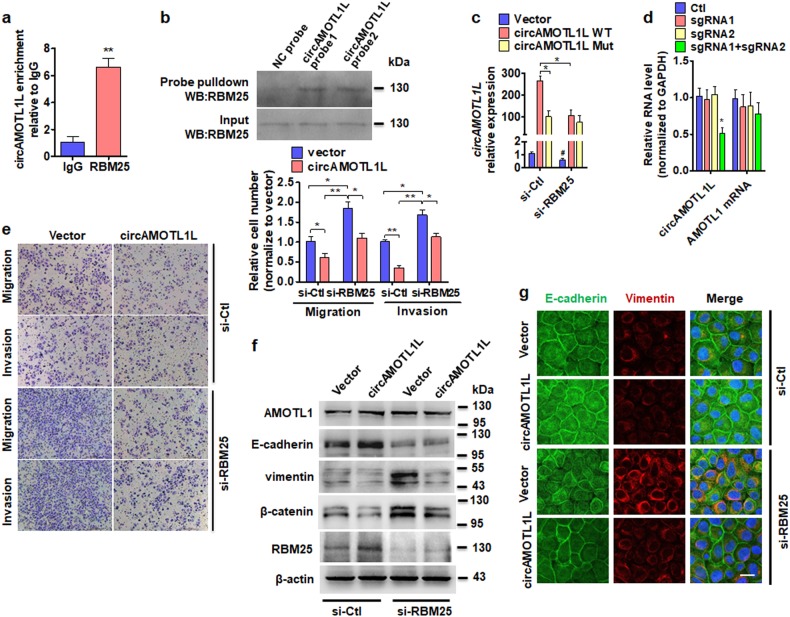
RBM25 and circAMOTL1L interact with each other and form a feedback regulation. **a** RNA immunoprecipitation (RIP) was performed with an anti-RBM25 antibody in lysates of PC3 cells stably transfected with circAMOTL1L, and then the anti-RBM25 immunoprecipitates were used to detect circAMOTL1L by qRT-PCR. **P<0.01 vs. IgG. **b** PC3 cells were co-transfected with expression vectors for circAMOTL1L and RBM25 for 24 h, and then cell lysates were pulled down with two different probes against circAMOTL1L. Western blot analysis detected RBM25. **c** The RBM25-binding sites were deleted in circAMOTL1L expression vector (circAMOTL1L Mut), and then these two expression vectors (WT or Mut) were co-transfected with RBM25-specific siRNA (si-RBM25) or si-Ctl into PC3 cells. qRT-PCR detected circAMOTL1L expression in PC3 cells. *P < 0.05 vs. circAMOTL1L WT + si-Ctl, ^#^*P* < 0.05 vs. empty vector + si-Ctl. **d** The RBM25-binding region in genomic DNA was deleted by two sgRNAs (sgRNA1+sgRNA2)-mediated CRISPR interference, qRT-PCR detected circAMOTL1L and AMOTL1 mRNA expression in PC3 cells. *P < 0.05 vs. sgRNA1 or sgRNA2. **e** PC3 cells were transfected with circAMOTL1L expression vector and RBM25-specific siRNA (si-RBM25) or their corresponding controls, and cell migration and invasion were evaluated by transwell migration and matrigel invasion assay. *P < 0.05 vs. empty vector, **P < 0.01 vs. circAMOTL1L overexpression. **f** Indicated proteins were examined in PC3 cells treated as in (**e**) by western blot analysis. **g** Immunofluorescence staining detected E-cadherin (green) and vimentin (red) in PC3 cells treated as in (**e**). Scale bars=20μ m. All graph bars are mean ± SEM of 3 independent experiments

To further define whether RBM25 mediates circAMOTL1L formation by directly binding to circAMOTL1L, we performed a RNA immunoprecipitation (RIP) with an antibody to RBM25 and detected the enrichment of circAMOTL1L. As shown in Fig. 7a, circAMOTL1L was enriched by at least 6-fold in the anti-RBM25 immunoprecipitates compared to those in IgG immunoprecipitates. Further, we used two specific probes for circAMOTL1L sequences to pull down RBPs interacting with circAMOTL1L and detected RBM25 in the co-precipitated proteins by Western blotting. The results showed that two circAMOTL1L-specific probes could effectively pull down RBM25 relative to the negative control probe (NC probe) (Fig. 7b). These results suggest that RBM25 binds directly to circAMOTL1L. Because silencing of RBM25 did not affect the stability of circAMOTL1L (Supplementary Fig. 9a), we sought to know how RBM25 regulates circAMOTL1L biogenesis by binding to its sequence. Previous studies have demonstrated that RBM25 could bind to poly-G sequences or the exonic splicing enhancer 5′-CGGGCA-3′ motif to regulate alternative splicing through interactions with the splicing machinery [39, 40]. Thus, we determined whether the binding site for RBM25 in the circAMOTL1L sequence is necessary for circAMOTL1L biogenesis. First, we mutated the RBM25-binding sequence in a pcDNA-circAMOTL1L vector and used qRT-PCR to detect circAMOTL1L expression. As shown in Fig. 7c and Supplementary Fig. 9b, expression level of circAMOTL1L was significantly reduced in PC3 cells transfected with the RBM25-binding site-mutated recombinant vector (Mutant) compared with that transfected with the pcDNA-circAMOTL1L vector containing wild-type RBM25-binding site (WT). Notably, knockdown of RBM25 also decreased significantly circAMOTL1L expression in PC3 cells transfected with the pcDNA-circAMOTL1L containing wild-type RBM25-binding site (WT). Next, we deleted the sequences that contain RBM25-binding site in genomic DNA by using a single-chain guide RNA (sgRNA) recognizing RBM25-binding site. Similarly, deletion of RBM25-binding sequence in genomic DNA reduced circAMOTL1L expression by 50% of the control but did not obviously have impact on AMOTL1 mRNA expression (Fig. 7d). Further, we detected the circAMOTL1L and RBM25 expression in human PCa tissues by using in situ hybridization of circAMOTL1L probe combined with immunostaining with the anti-RBM25 antibody and found that circAMOTL1L and RBM25 were mainly co-localized in the same cells (Supplementary Fig. 9c). Together, these data suggest that RBM25 binds directly to circAMOTL1L and induces its biogenesis.

To further assess the influence of RBM25 on circAMOTL1L-regulated cell migration and invasion, we performed the transwell membrane and matrigel invasion assays with PC3 cells transfected with circAMOTL1L-expressing vector, RBM25-specific siRNA (si-RBM25) or their corresponding negative control. As shown in Fig. 7e, silencing of RBM25 in PC3 cells significantly promoted cell migration and invasion compared with that transfected with the negative control RNA (si-Ctl), and reversed the inhibitory effect of circAMOTL1L overexpression on cell migration and invasion. Western blot analysis also revealed that knockdown of RBM25 reduced E-cadherin expression and elevated the expression levels of vimentin and β-catenin, but not AMOTL1. However, the effect of RBM25 knockdown on the expression of E-cadherin, vimentin and β-catenin could be partly reversed in the circAMOTL1L-overexpressing PC3 cells (Fig. 7f). Consistently, the similar results were obtained by immunofluorescent staining of circAMOTL1L-overexpressed and RBM25-depleted PC3 cells for E-cadherin and vimentin, showing an opposing effect between the RBM25 knockdown and circAMOTL1L overexpression (Fig. 7g). Interestingly, overexpression of circAMOTL1L increased the expression level of RBM25 protein (Fig. 7f, lane 2 versus lane 1), while circAMOTL1L knockdown dramatically decreased RBM25 expression (Supplementary Fig. 9d). Collectively, these findings suggest that RBM25 and circAMOTL1L form a feedback loop to co-operatively regulate the EMT-related gene expression in PCa cells.

## Discussion

Previous studies have shown that the tumor suppressor p53 regulates the epithelial–mesenchymal transition (EMT) and plays critical roles in cancer metastasis [41]. In about half of all human cancers, p53 is either lost or mutated [42]. Loss of wild-type p53 function affects cell cycle checkpoint controls and apoptosis [43] and mutation of the p53 gene is responsible for development and progression of many cancers [42, 44]. In recent years, circRNAs and miRNAs were reported to be important components of the cellular signaling network that regulates the EMT program [15, 20]. In PCa, however, it has not been investigated whether and how p53 regulates EMT through circRNAs and miRNAs. In the present study, we show for the first time that the p53-RBM25-mediated circAMOTL1L-miR-193a-5p pathway is a new link between p53 and EMT by regulating the expression of EMT-related genes in PCa cells.

CircRNAs have been reported to be dysregulated in diverse cancer types, such as bladder cancer, hepacellular carcinoma, esophageal squamous cell carcinoma and basal cell carcinoma [45]. It is widely accepted that these differentially expressed circRNAs may have important functions in regulation of gene expression [25]. Despite recent discoveries regarding disease-related circRNAs, little is known about the biogenesis of circRNAs and the underlying molecular mechanism of circRNA-mediated gene regulation during PCa development. In this study, we present the first evidence showing a comprehensive expression profile of differentially expressed circRNAs in high-grade PCa and low-grade PCa within the same PCa sample. Thousands of circRNAs were detected to be differentially expressed between these two PCa tissues. Among these, circAMOTL1L (hsa_circRNA_000350) was significantly downregulated in high-grade PCa tissues compared with low-grade PCa and BPH. A recent study shows that circ-Amotl1, which is produced by exon 3 of angiomotin-like 1 gene (Amotl1), is highly expressed in human cardiac tissue and can reduce apoptosis induced by doxorubicin (Dox) and enhance cardiac repair through binding to and activating AKT phosphorylation and nuclear localization [23]. Here, we found that circular RNA synthesised in PCa cells is different from that in cardiac tissue, this circRNA, termed circAMOTL1L, was derived from circularization of the exon-2 and exon-3 of the Amotl1 gene. It has been known that AmotL1 is a novel partner of the N-cadherin protein complex that comprises tight junctions, which form the apical junctional structures involved in controlling paracellular permeability and cell polarity [46, 47]. Moreover, a previous report revealed that AmotL1 is critical for cadherin-11-mediated cell migration and may be involved in promoting migration in prostate cancer [46]. Based on these observations, it can be speculated that Amotl1 gene-derived circAMOTL1L might exert functional roles in the regulation of EMT and PCa metastasis.

Epithelial-to-mesenchymal transition (EMT) is regarded as an important step in cancer metastasis. The primary PCa tumor epithelial cells need to undergo EMT to become invasive and to metastasise [20]. To explore the mechanism underlying circAMOTL1L-mediated EMT, we investigated whether circAMOTL1L affects migration and invasion of PCa cells through downregulating E-cadherin and upregulating mesenchymal markers. As expected, circAMOTL1L overexpression in PC3 and DU145 cells obviously reduced, while knockdown of circAMOTL1L by siRNA markedly increased PCa cell migration and invasion. This is consistent with downregulation of circAMOTL1L expression in PCa tissue specimens, suggesting that decreased circAMOTL1L facilitates PCa cell migration and invasion, contributing to PCa aggressiveness. Using circAMOTL1L-overexpressed or depleted PC3 cells, we further demonstrated that circAMOTL1L overexpression increased E-cadherin expression and decreased expression of vimentin and β-catenin. In contrast, silencing of circAMOTL1L in PC3 cells reduced E-cadherin and increased vimentin and β-catenin protein levels. These results indicated that circAMOTL1L deficiency enhances the migration and invasion of PCa cells by downregulation of E-cadherin and upregulation of mesenchymal cadherins.

Within the regulatory networks governing gene expression, circRNAs have been shown to exert multiple functions by acting as cytoplasmic miRNA sponges, RNA-binding proteins, and nuclear transcriptional regulators [12]. For example, circular Foxo3 regulates the expression of its parent gene, Foxo3, by acting as a sponge for endogenous miRNAs [48]. EIciRNAs, an exon-intron circRNA, predominantly localize in the nucleus, interact with U1 snRNP and promote transcription of their parental genes [49]. We found that circACTA2 mediates NRG-1-ICD regulation of α-SMA expression in HASMCs and regulates cell contraction [38]. However, it remains unknown regarding the role of circAMOTL1L in the regulation of expression of its parental gene or EMT-related genes.

In this study, we shows that circAMOTL1L sponges miR-193a-5p, which was found to be upregulated in PCa tissue and involved in the resistance of PCa cells to docetaxel-induced apoptosis [19]. Considering the fact that circAMOTL1L overexpression increased, while knockdown of circAMOTL1L reduced E-cadherin expression, we speculated that E-cadherin gene might be a direct target of miR-193a-5p in PCa cells. Indeed, the bioinformatics analysis showed that Pcdha gene cluster, which belongs to a subset of the cadherin superfamily members that mediate cell-cell adhesion, contains highly conserved miR-193a-5p-binding site in their 3' UTR. Although little is known about Pcdh molecular functions in the context of tumor biology, multiple lines of evidence suggest that clustered Pcdh plays critical roles in tumor growth and metastasis [10, 11]. It has been reported that overexpression of individual Pcdhg cDNAs reduces tumor cell growth by inhibiting the Wnt pathway [50], and that the ubiquitously-expressed γ-Pcdh-C3 isoform is silenced in colorectal cancer cells, and its overexpression reduces growth of these cells [10]. Additionally, Pcdhga11 and Pcdhb genes were shown to be aberrantly methylated in astrocytomas [51] and neuroblastoma [52]. These data suggest that clustered Pcdh may act as tumor suppressors.

A number of circRNAs involved in EMT are regulated by the RNA-binding protein Quaking (QKI)[20]. It has been known that QKI regulates pre-mRNA splicing through binding to sites flanking circRNA-forming exons [20, 53]. However, our results show that knockdown of QKI did not affect the expression of circAMOTL1L in PCa cells. This is most likely because biogenesis of different circRNAs is regulated by different RNA-binding proteins in different cell and tissue types. For example, RBM20 is crucial for the formation of a subset of circRNAs that originate from the I-band of the titin gene, and these circRNAs are dynamically regulated in dilated cardiomyopathy [26]. NF90/NF110 promote circRNA production via its double-stranded RNA-binding activity in viral infection [54]. Our previous study revealed that NRG-1-ICD induces circACTA2 formation via recruiting IKAROS family zinc finger (IKZF1) to the first intron of *α-SMA* gene [38]. A recent report revealed that RBM25 regulates a large fraction of alternatively spliced exons throughout the human genome via its interaction with the exonic splicing enhancer, CGGGCA sequence, which is located within exon [39, 40]. Here we found that circAMOTL1L contains two binding sites of RBM25. Both the RNA immunoprecipitation (RIP) with an antibody to RBM25 and RNA pull-down assays demonstrated that RBM25 binds directly to circAMOTL1L and induces its biogenesis. Further, we showed that deletion of RBM25-binding sequence or silencing of RBM25 in PC3 cells significantly increased cell migration and invasion. Although circRNAs derived from different genes are formed by different types of alternatively splicing, bioinformatic analysis revealed shared features of circularized exons, including long bordering introns that contain complementary ALU repeats [28]. In this study, we identified that exons 2 and 3 of AMOTL1 gene, which form circAMOTL1L, comprise, respectively, 26,450-bp upstream and 21,218-bp downstream flanking intronic sequences. These long flanking sequence with complementary Alu repeats facilitates the formation of a circRNA from these exons.

These findings raise the important questions about how RBM25 expression is regulated in PCa cells. Because it is known that wild-type p53 suppresses EMT in PCa cells and that metastatic progression of cancers involves the downregulation of p53 expression [42], we examined p53 expression in PCa and BPH tissues. As expected, p53 protein levels were markedly reduced in PCa tissue compared with BPH. Importantly, our results show that RBM25 is a direct transcriptional target of p53, as evidenced by luciferase reporter gene assays and ChIP analysis. Moreover, depletion of p53 in PC3 cells dramatically decreased the expression of RBM25, circAMOTL1L, Pcdha8, and E-cadherin. Together, our data show that p53 regulates EMT-related gene expression via RBM25-mediated circAMOTL1L formation. These findings have linked p53-RBM25-mediated circAMOTL1L-miR-193a-5p-Pcdha regulatory axis to EMT in metastatic progression of PCa. Targeting this newly identified regulatory axis provides a potential therapeutic strategy for aggressive PCa.

## Materials and Methods

The detailed procedures of plasmid construction, cell transfections, cell metastasis assays, antibody and immunoblot, microarray analysis, xenograft animal model, RNA isolation, qRT-PCR, chromatin immunoprecipitation (ChIP)-qPCR, immunofluorescence staining, in situ hybridization, oligo pulldown, morphometry and histology, luciferase reporter assay, northern blotting, RNA immunoprecipitation (RIP) assays, CRISPR/Cas9-mediated genomic editing, p53 knockout and RNA-Seq analysis as well as key reagents are described in Supplementary Experimental Procedures.

### Clinical samples and microarray

Patients (median age 65 years, range 52 to 79) underwent radical prostatectomy for localized PCa (*n* = 62) and benign prostatic hyperplasia (*n* = 35) underwent transurethral resection of the prostate (TURP) at the Department of Urology, the Second Hospital of Hebei Medical University, China from July 2014 to October 2017. No treatment was administered prior to surgery. All the tissue specimens were confirmed by two experienced pathologists. Pathological grading was judged by Gleason points-scoring system, Gleason score ≥8 (high PCa, *n* = 28) and Gleason score <8 (low PCa, *n* = 34). The patient characteristics are summarized as previously described [19]. The study protocol was approved by the Ethics Committee of Second Hospital of Hebei Medical University and Verbal consent was obtained from each patient. Microarray hybridization analysis of circRNA expression in five pairs of PCa samples with areas of high-grade disease (Gleason>8) or areas of low-grade (Gleason<6) was performed according to the manufacturer’s protocol (Arraystar, Inc., Rockville, MD, USA).

### Cell culture

PC3 (CRL-1435; ATCC), LNCaP (CRL-1740; ATCC), and DU145 cells (HTB-81; ATCC) were grown and maintained in RPMI 1640 medium (Gibco, USA) containing penicillin (100 units/ml) and streptomycin (100 µg/ml). RWPE-1 cells (CRL-11609; ATCC) were maintained in K-SMF medium (Life Technologies, USA) supplemented with 5 ng/mL epidermal growth factor (EGF) and 50 μg/mL bovine pituitary extract. Cultures were incubated in a humidified environment at 5% CO_2_ and 37 °C.

### Statistical analysis

All the data are presented as means ± standard errors of the means (SEM). Differences between two groups were assessed using an analysis of variance, followed by Student's t-test. A *P* value <0.05 was considered statistically significant. The statistical analysis was performed using Prism 5 software (GraphPad Software, San Diego, CA, USA). For xenograft experiments, sample sizes were calculated using the standard deviations from preliminary experiments and the expected difference between two groups. Other sample sizes were equal to or greater than the recommended minimum sample sizes in previous publications.

## Electronic supplementary material

Supplemental Experimental Procedures

Supplementary Figure 1–9

Supplementary Table 1

Supplementary Table 2

Supplementary Table 3

Supplementary Table 4

Supplementary Table 5
