# Supplementary material for: Dysregulation of p53-RBM25-mediated circAMOTL1L biogenesis contributes to prostate cancer progression through the circAMOTL1L-miR-193a-5p-Pcdha pathway
Source: Oncogene. 2018 Dec 7;38(14):2516–32. doi: 10.1038/s41388-018-0602-8 (PMC6484770; doi:10.1038/s41388-018-0602-8)
Supplement: Supplementary file 1 — Supplemental Experimental Procedures [file 41388_2018_602_MOESM1_ESM.pdf]

Supplemental Experimental Procedures

Microarray

Place a freshly dissected cross-section prostate tissue (5mm thick) on to a pre-labeled tissue base mold. Cover the entire tissue block with Surgipath FSC 22 Frozen Section Embedding Medium (Leica). Slowly place the base mold containing the tissue block into liquid nitrogen till the entire tissue block is submerged into liquid nitrogen to ensure tissue is frozen completely. Store the frozen tissue block at -80°C until ready for sectioning. Transfer the frozen tissue block to a cryotome cryostat (-20 °C) prior to sectioning and allow the temperature of the frozen tissue block to equilibrate to the temperature of the cryotome cryostat. Section the frozen tissue block into 8 μm using the cryotome. Place the tissue sections onto glass slides suitable for HE staining and immunohistochemistry. The tissue specimens were confirmed by two experienced pathologists. Pathological grading was judged by Gleason points-scoring system, Gleason score ≥8 and Gleason score <8. Total RNAs were extracted and digested with RNase R (Epicentre, Inc., Madison, WI, USA) to remove linear RNAs and thus enrich circRNAs. The enriched circRNAs were amplified and transcribed into fluorescent cRNA using a random priming method (Arraystar Super RNA Labeling Kit; Arraystar). The labeled cRNAs were purified using an RNeasy Mini Kit (Qiagen, Venlo, Netherlands). The concentration and specific activity of the labeled cRNAs (pmol Cy3/μg cRNA) were measured using a NanoDrop ND-1000 (Thermo Scientific, Waltham, MA, USA). One microgram of each labeled cRNA was fragmented by adding 5 μl of 10× Blocking Agent and 1 μl of 25× Fragmentation Buffer; after heating the mixture at 60 °C for 30 min, 25 μl of 2× Hybridization Buffer was added to dilute the labeled cRNA. Next, 50 μl of hybridization solution was dispensed into the gasket slide and assembled to the circRNA expression microarray slide. The slides were incubated for 17 h at 65°C in an Agilent hybridization oven (Agilent Technologies, Santa Clara, CA, USA). The hybridized arrays were washed, fixed, and scanned using the Agilent Scanner G2505C. Agilent Feature Extraction software (version 11.0.1.1) was used to analyze the acquired array images. Quantile normalization and subsequent data processing were performed using the GeneSpring GX v11.5.1 software package (Agilent Technologies). The raw and normalized microarray data have been deposited in the Gene Expression Omnibus database under accession number GSE113180.

Cell transfections

All cells were transfected using Lipofectamine 2000 (Invitrogen/Life Technologies) according to the manufacturer's protocol. Small interfering RNAs (siRNAs) targeting the indicated gene sequences (Supplementary Table 2) were designed and synthesized by GenePharma (Shanghai, PR China). Expression plasmids for circAMOTL1L, p53 and RBM25 are described below (vector construction).

Cell migration, invasion, and wound-healing assays

Approximately 3 × 10^4 PC3 or DU145 cells per well were plated in the upper chambers of 24-well Transwell plates (#3422; Corning) overnight and transfected with circ-pcDNA3.1-circAMOTL1L (pcDNA-circAMOTL1L) or circ-pcDNA3.1 plasmids. After 6 h, the medium was replaced with complete medium (10% fetal bovine serum, FBS), and cells were cultured for an additional 18 h. Cells remaining on the upper filter were removed; those that passed through the Transwell filter were stained using hematoxylin–eosin (HE). Images were taken from six random optical fields (200×) per filter, and cell numbers were quantified using a Leica microscope (DM6000B; Leica Microsystems, Heerbrugg, Switzerland) and digitized using LAS V.4.4 software (Leica). To study cell invasion, Transwell membranes were coated with matrigel (Sigma-Aldrich, St. Louis, MO, USA) prior to plating cells. The matrigel served as a basement membrane barrier that cells would have to destroy before invading the lower chamber. After a 24-hour treatment, HE staining and cell counting were performed as above. For the wound-healing assay, PC3 or DU145 cells were grown on 12-well plates and transfected with pcDNA-circAMOTL1L or circ-pcDNA3.1 plasmids. Confluent cell layers were scraped using a 200-μL pipette tip, washed twice with PBS, and cultured in media containing 10% serum for 24 h. Images were acquired using an Olympus microscope (CKX41; Olympus Corporation, Tokyo, Japan) equipped with a camera (EOS 600D; Canon Inc., Tokyo, Japan) immediately (0 h) and at 24 h after wounding.

Plasmid and lentivirus expression vector constructs

In order to overexpress a circular RNA with a seamless connection between its 5' and 3' ends without redundant nucleotides, we constructed a circ-pcDNA3.1 vector with a reverse repeat sequence combined with 5' donor splice sequences and 3' acceptor splice sequences (Supplementary Table 2). Between the donor splice site and acceptor splice site, we inserted a EcoNI enzyme site with sequences of 5'-CCTCAG^{v}CTAGG-3' and a PmlI enzyme site with sequences of 5'-CAC ^{v} GTG-3'. The full length sequence of circAMOTL1L was amplified from cDNA and inserted to EcoNI (NEB) and PmlI (NEB)-digested circ-pcDNA3.1 with one-step cloning (C112-02; Vazyme Biotech Co., Ltd.). Sequences of WT or mutant circAMOTL1L or the 3' untranslated region (UTR) sequences of *Pcdha8* containing WT or mutated miR-193a-

5p binding site were inserted into the pmir-GLO Dual-Luciferase miRNA Target Expression Vector (Promega Corp., Madison, WI, USA). All plasmids were sequenced for confirmation. All oligos used as primers for plasmid construction and probe preparation, siRNA, and biotinylated-oligos are listed in Supplementary Table 2. Lentivirus encoding p53 (LV-p53), circAMOTL1L (LV-circAMOTL1L), anti-miR-193a-5p (LV-anti-miR-193a-5p) and LV control (LV-Ctl) were entrusted to Hanbio, shanghai. The expression plasmid of RBM25 was created by inserting human RBM25 cDNA into the pcDNA3.1 vector; p53 cDNA was amplified from LV-p53 and then inserted into the pcDNA3.1 vector.

Xenograft animal model

All animal studies were approved by the Institutional Animal Care and Use Committee of Hebei Medical University (approval ID: HebMU 20080026), and all efforts were made to minimize suffering. Xenograft model was established as described previously(1). In brief, male BALB/c nude mice at 4–6 weeks of age (18–22 g) were purchased from Vital River Laboratory Animal Technology Co., Ltd. (Beijing, china). 5×10^6 PC3 cells stably expressing miR-193a-5p, circAMOTL1L or both were harvested by trypsinization, resuspended in 0.2 mL PBS, and mixed with 50% Matrigel (Collaborative Research Inc., Bedford, MA, USA); and then this suspension was injected subcutaneously into the right dorsal flanks of nude mice. The length and width of mouse tumor were measured twice a week with calipers. Then the following formula was used to calculate tumor volume ($volume=[(length\times width^2)/2]$). At the end of the experiment, the mice were euthanized by Carbon dioxide asphyxiation. At last, the tumor tissues were fixed in 4% formalin solution or flash-frozen in liquid nitrogen immediately, and stored at -80 °C until further use.

Isolation of mRNA and real time PCR

Clinical tumor samples and xenograft tumors were homogenized with a gentle MACSTM Dissociator (Miltenyi Biotec, Bergisch Gladbach, Germany), and cultured cells were lysed using QIAzol Lysis Reagent (79306). Total RNA was extracted from the above samples with a miRNeasy Mini Kit (217004; Qiagen) according to the manufacturer's instructions. RNA quality was determined using a NanoDrop 2000. For miRNA, reverse transcription and quantitative real-time (qRT)-PCR were performed using the miScript II RT Kit (218161) and miScript SYBR Green PCR Kit (Catalog no. 218073) with primers for specific miRNAs (Supplementary Table 2) and the internal control U6, according to the manufacturer's protocol. For large mRNA, cDNA was synthesized using a M-MLV First Strand Kit (Life Technologies) with random hexamer primers. mRNAs or circRNAs were subjected to qRT-PCR using the Platinum SYBR

Green qPCR Super Mix UDG Kit (Invitrogen) and the ABI 7500 FAST system (Life Technologies). Relative transcript expression levels were normalized to GAPDH and calculated using the $2^{-\Delta\Delta Ct}$ formula as previously described(2). Supplementary Table 2 summarizes the primer sequences.

Western blot analysis

Western blotting was carried out using a standard protocol as previously described(2,3). The total protein from tumor tissues were extracted by homogenization, and cultured cells were lysated with lysis buffer (1% Triton X-100, 150 mM NaCl, 10 mM Tris-HCl, pH 7.4, 1 mM EDTA, 1 mM EGTA, pH 8.0, 0.2 mM Na_3VO_4, 0.2 mM phenylmethylsulfonyl fluoride, and 0.5% NP-40). Equal amounts of protein were separated on SDS-PAGE, and electrotransferred to a PVDF membrane (Millipore). Membranes were blocked with 5% milk in TTBS for 2 h at room temperature and incubated overnight with primary antibodies at 4 °C. Antibodies that were used are as follow: anti-E-cadherin (1:1000, ab1416), anti-vimentin (1:1000, sc-6260), or anti-β-actin (1:1000, sc-47778), anti-β-catenin (1:500, ab2365), anti-RBM25 (1:500, sc-374271), anti-Pcdha8 (1:500, ab170788), anti-AMOTL1 (1:500, ab171976), anti-p53 (1:1000, sc-126). Membranes were then incubated with the HRP-conjugated secondary antibody (1:5000, Rockland) for 1 h at room temperature. The blots were treated with the Immobilon™ Western (Millipore), and detected by ECL (enhanced chemiluminescence) Fuazon Fx (Vilber Lourmat). Images were captured and processed by FusionCapt Advance Fx5 software (Vilber Lourmat). All experiments were replicated three times.

Morphometry and histology

Human PCa and BPH tissues were fixed in 10% neutral buffered formalin solution and then processed for routine embedding in paraffin. Ten consecutive 5-μm-thick sections were prepared for hematoxylin and eosin staining. PCa cross-section images were acquired using a Leica digital pathology scanner (Aperio CS2, Switzerland) and digitized with Image-Scope-Rev-v12.1.0.5029 (Leica).

Fluorescence *In situ* hybridization

In situ hybridization was performed as described previously(3). In brief, cells were fixed in 4% paraformaldehyde for 5 min at room temperature and subsequently washed with PBS. Paraffin-embedded cross-sections (5-μm-thick) from clinical tissues were deparaffinized and rehydrated for *in situ* hybridization according to the miRCURY LNA™ microRNA ISH Optimization Kit manual (Exiqon, Vedbæk, Denmark). Hybridization with fluorescence-labeled circAMOTL1L or miR-193a-5p probes

(Supplementary Table 2) in hybridization buffer (Exiqon) was performed during a 1-h incubation at 55°C in a thermoblock (Labnet, Edison, NJ, USA). After stringent washing with SSC buffer, samples were treated with DAPI (157574, MB biomedical) for nuclear counterstaining. Images were captured using a confocal microscope (DM6000 CFS; Leica) and processed using LAS AF software. Further images were acquired using a Leica DM6000B microscope and digitized using LAS V.4.4 software.

Immunofluorescence staining

Immunofluorescence staining was performed as described previously(2-4). In brief, five-micrometer paraffin-embedded cross-sections of tissues or cultured PC3 cells were subjected to immunofluorescence staining. Sections were deparaffinized with xylene, rehydrated, and pre-incubated with 10% normal goat serum (710027, KPL, USA) followed by incubation with the following primary antibodies: anti-E-cadherin (20874-1-AP), anti-vimentin (10366-1-AP), anti-Pcdha8 (ab170788), anti-β-catenin (ab2365), anti-RBM25 (sc-374271). Sections were subsequently treated with the following secondary antibodies: fluorescein-labeled antibody to rabbit IgG (021516; KPL, USA) and rhodamine-labeled antibody to mouse IgG (031806; KPL), or fluorescein-labeled antibody to mouse IgG (021815; KPL) and rhodamine-labeled antibody to rabbit IgG (031506; KPL). In each experiment, DAPI (157574; MB Biomedical) was used for nuclear counterstaining. Images were captured using a confocal microscope (DM6000 CFS; Leica) and processed using LAS AF software.

Biotinylated-oligo pulldown for RNAs or circAMOTL1L-binding proteins

To detect interactions between circAMOTL1L and miRNAs, a biotin-oligo pulldown was performed as previously described, with some modifications(5). Briefly, log-phase cells were cross-linked with 1% glutaraldehyde or formaldehyde in PBS for 10 min at room temperature; the cross-linking was then quenched with 0.125 M glycine for 5 min. The cells were pelleted and resuspended in lysis buffer (50 mM Tris, pH 7.0, 10 mM EDTA, 1% SDS and 1 mM DTT, complete protease inhibitor, and 0.1 U/μl RNase inhibitor were freshly added) on ice for 10 min; these suspensions were then sonicated until most chromatin had solubilized and DNA fragment sizes were approximately 100–500 bp. Chromatin was diluted in a two-fold volume with hybridization buffer (750 mM NaCl, 1% SDS, 50 mM Tris, pH 7.0, 1 mM EDTA, 15% formamide, 1 mM DTT, protease inhibitor, and 0.1 U/μl RNase inhibitor). Biotin-DNA oligos (100 pmol) were added to 3 ml of diluted chromatin, and solutions were mixed by end-to-end rotation at 37 °C for 4 h. M-280 Streptavidin Dynabeads (Life Technologies) were washed three times in lysis buffer, blocked with 500 ng/μl yeast total RNA for 1 h at room temperature, and again washed three times in lysis buffer prior to resuspension. Next, 100 μl of

washed/blocked Dynabeads were added per 100 pmol of biotin-DNA oligos, and the mixture was then rotated for 30 min at 37°C. Beads were captured by magnets (Life Technologies) and washed five times using a 40×volume of wash buffer (2×SSC, 0.5% SDS, and 0.1 mM DTT and PMSF). RNA was then eluted from the beads using elution buffer (50 mM Tris, pH7.0, 1% SDS). Protein was eluted with a cocktail of 100 ug/ml RNase A (Solarbio), 0.1 U/ul RNase H (Thermo Scientific), and 100 U/ml DNase I (Thermo Scientific) at 37 °C for 30 min. Differentially interacted proteins were identified using Mass Spectrometry. Analysis was carried out on the Ultrafle Xtreme LC-MS/MS mass spectrometer at Kangchen in Shanghai, China. circAMOTL1L-interacted proteins were separated on a 10% SDS-PAGE and analyzed by Western blotting with a RBM25 antibody.

Northern blotting

Northern blot analysis of circRNAs or linear RNAs was performed as described previously(2). PCR primers including T7 promoter sequences were designed to target the linear transcript of AMOTL1, or the circAMOTL1L transcript exclusively (AMOTL1: 603 bp; circAMOTL1L: 421 bp). Supplementary Table 2 summarizes the primer sequences. The *in vitro* transcription using 10 μg unpurified PCR-template was performed using DIG Northern Starter Kit (Roche; 12039672910) including subsequent DNase I digestion of the template according to the manufacturer's instruction. Total RNA was mixed with RNA loading dye and loaded onto 2% agarose gel in MOPS buffer. Subsequently, the gel was equilibrated in 20 × SSC for 2 × 15 min and gels are blotted by capillary transfer with 20 × SSC overnight. The membranes were UV cross-linked and prehybridized with DIG Easy Hyb for 30 min with gentle agitation in an appropriate container. For hybridization, the membranes were then incubated with 100 ng/ml DIG-labelled RNA antisense probe in hybridization buffer at 68 °C overnight. After washing (two times for 5 min with 2 × SSC, 0.1% SDS followed by two times for 15 min with 0.1 × SSC, 0.1% SDS at 68 °C), the membranes were detected according to the manual (Roche, DIG Northern Starter Kit).

RNA immunoprecipitation (RIP) assays

PC3 cells were co-transfected with pcDNA3.1-RBM25 and pcDNA3.1-circAMOTL1L for 48 h, and then cells were used to conduct RIP experiments using a RBM25 antibody (sc-374271) or IgG, and the Dynabeads™ Protein G Immunoprecipitation Kit (10007D, Thermo Fisher) according to the manufacturer's instructions. The RNA fraction isolated by RIP was quantified by NanoDrop 2000 (Thermo-Fisher). The cDNA was synthesized using a M-MLV First Strand Kit (Life Technologies) with random hexamer primers. The RIP' circAMOTL1L was subjected to qRT-PCR using the Platinum SYBR

Green qPCR Super Mix UDG Kit (Invitrogen) and the ABI 7500 FAST system (Life Technologies).

Luciferase assay

PC3 cells were maintained as previously described(1). For luciferase assays, PC3 cells were co-transfected with a miR-193a-5p mimic (Gene pharma; Shanghai) or NC mimic (200 pmol) combined with 100 ng of luciferase reporter or an empty vector; and PC3 cells were also transfected with a luciferase-harboring RBM25 promoter construct and pcDNA3.1-p53 using Lipofectamine 2000 (Invitrogen) according to the manufacturer's protocol. Luciferase activity was measured using a Dual-Glo Luciferase Assay System (Promega, Madison, WI) with a Flash and Glow (LB955, Berthold Technologies) reader 24 h after transfection. The specific activity was expressed as the relative activity ratio of firefly to Renilla luciferase. All promoter constructs were evaluated in a minimum of three separate wells per experiment.

Chromatin immunoprecipitation (ChIP) assay

The chromatin immunoprecipitation (ChIP) assay was performed as described previously(2). In brief, PC3 cells were treated with 1% formaldehyde for 10 min to cross-link proteins with DNA. The cross-linked chromatin was then prepared and sonicated to an average size of 400–600 bp. The samples were diluted 10-fold and then precleared with protein A-agarose/salmon sperm DNA for 30 min at 4 °C. The DNA fragments were immunoprecipitated overnight at 4 °C with anti-p53, or anti-IgG (as negative control) antibodies. After cross-linking reversal, p53 occupancy on the RBM25 promoter was examined. Results were determined by qRT-PCR. ChIP primer sequences were summarized in Supplementary Table 2.

Deletion of a target gene by CRISPR/Cas9 technology

For the CRISPR/Cas9 assay, sgRNA design and cloning into retroviral construct were carried out as described previously(2,6). In brief, sgRNAs were constructed by annealing each pair of oligos (Supplementary Table 2) and ligating them to BsaI-linearized SaCas9 (Plasmid#61591, Addgene). The vectors were confirmed by sanger sequence. PC3 cells were seeded in 6-well plates at a density of 2×10^5 cells per well. After 24 h, the cells were transiently transfected with 2 μg CRISPR/Cas9 plasmid using Lipofectamine 2000 (Life Technologies). Genomic DNA and total RNA were extracted after 72 h of transfection using DNA Extraction Kit (Omiga) or QIAzol Lysis Reagent (79306). PCR was conducted to amplify the targeting region by using DNA template. The expression of circAMOTL1L was detected by qRT-PCR.

p53 knockout and RNA-Seq analysis

The p53 gene in PC3 cells was knocked out (p53-KO) by CRISPR/Cas9 technique as described previously(7). In brief, PC3 cells were transfected with constructs expressing Cas9-D10A (Nickase) and control sgRNAs or sgRNAs targeting p53 exon 3 (sc-437281 for control; sc-416469-NIC for targeting p53). After 48 h of transfection, cells were suspended, diluted and re-seeded to make sure single clone formation. GFP positive clones were picked up, and the expression of p53 in each single clone was evaluated by Western blotting with p53 (sc-126) antibody. The stable clones were seeded again in 10-cm plates at 3–5 days after culture, when the cells reached 50% confluency, the cells were washed with ice-cold PBS twice and RNA extraction was performed with the miRNeasy Mini Kit (217004; Qiagen) according to the manufacturer's instructions. RNA-seq analysis was performed at KangChen Bio-tech (Shanghai, China). Briefly, total RNA was quantified using a NanoDrop ND-1000 instrument. 1~2 μg of total RNA was used to prepare the sequencing library using KAPA Stranded RNA-Seq Library Prep Kit (Illumina). The completed libraries were qualified with Agilent 2100 Bioanalyzer and sequenced by using Illumina HiSeq 4000 instrument. Raw sequencing data were analyzed using Solexa pipeline v1.8 (Off-Line Base Caller software, v1.8). Sequence quality was examined using the FastQC software. The trimmed reads (trimmed 5',3'-adaptor bases using cutadapt) were aligned to genome reference sequences using Hisat2 software (v2.0.4). The transcript abundances for each sample were estimated with StringTie (v1.2.3), and the FPKM value for gene and transcript level was calculated with R package Ballgown (v2.6.0). To further analyze the differentially expressed transcripts in a more reliable interval, the following filter strategies were applied: 1) the average of FPKM in either sample group > 0.1; 2) the fold change between Ctl group and p53 group >2; 3) p value between Ctl group and p53 group <0.01. RNA-seq data have been deposited into NCBI Gene Expression Omnibus (GEO) with accession number GSE113180.

Supplemental references

1. Yang Z, Chen JS, Wen JK, Gao HT, Zheng B, Qu CB*, et al.* Silencing of miR-193a-5p increases the chemosensitivity of prostate cancer cells to docetaxel. Journal of experimental & clinical cancer research : CR **2017**;36:178
2. Sun Y, Yang Z, Zheng B, Zhang XH, Zhang ML, Zhao XS*, et al.* A Novel Regulatory Mechanism of Smooth Muscle alpha-Actin Expression by NRG-1/circACTA2/miR-548f-5p Axis. Circulation research **2017**;121:628-35
3. Yang Z, Zheng B, Zhang Y, He M, Zhang XH, Ma D*, et al.* miR-155-dependent

regulation of mammalian sterile 20-like kinase 2 (MST2) coordinates inflammation, oxidative stress and proliferation in vascular smooth muscle cells. Biochimica et biophysica acta **2015**;1852:1477-89

4. Ma D, Zheng B, Suzuki T, Zhang R, Jiang C, Bai D*, et al.* Inhibition of KLF5-Myo9b-RhoA Pathway-Mediated Podosome Formation in Macrophages Ameliorates Abdominal Aortic Aneurysm. Circulation research **2017**;120:799-815
5. Chu C, Qu K, Zhong FL, Artandi SE, Chang HY. Genomic maps of long noncoding RNA occupancy reveal principles of RNA-chromatin interactions. Molecular cell **2011**;44:667-78
6. Ran FA, Cong L, Yan WX, Scott DA, Gootenberg JS, Kriz AJ*, et al.* In vivo genome editing using Staphylococcus aureus Cas9. Nature **2015**;520:186-91
7. Wang D, Kon N, Lasso G, Jiang L, Leng W, Zhu WG*, et al.* Acetylation-regulated interaction between p53 and SET reveals a widespread regulatory mode. Nature **2016**;538:118-22
