# Supplementary material for: Dysregulation of p53-RBM25-mediated circAMOTL1L biogenesis contributes to prostate cancer progression through the circAMOTL1L-miR-193a-5p-Pcdha pathway
Source: Oncogene. 2018 Dec 7;38(14):2516–32. doi: 10.1038/s41388-018-0602-8 (PMC6484770; doi:10.1038/s41388-018-0602-8)
Supplement: Supplementary file 2 — Supplementary Figure 1–9 [file 41388_2018_602_MOESM2_ESM.pdf]

Supplementary Material

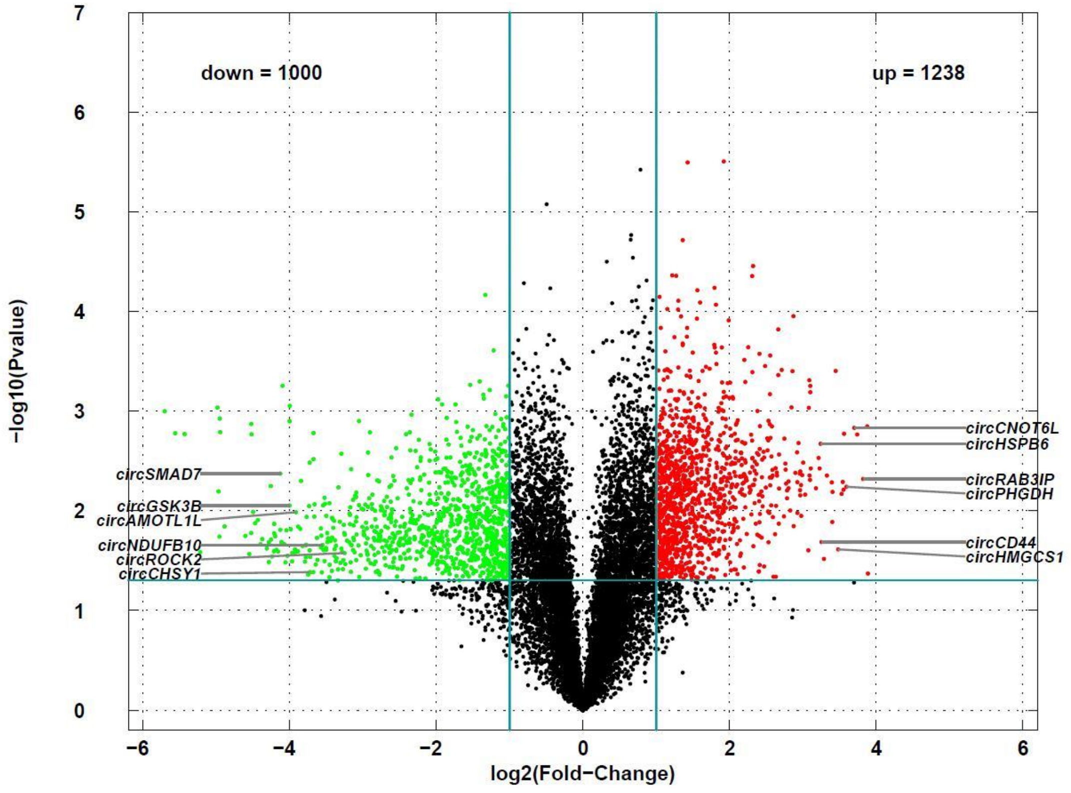

Supplementary Figure 1. Volcano plots are using for visualizing differential expression between two different PCa tissues, with some being labelled among significantly changed genes. The vertical lines correspond to 2.0 fold up (red) and down (green), respectively, and the horizontal line represents a P-value of 0.05 (− log10 scaled).

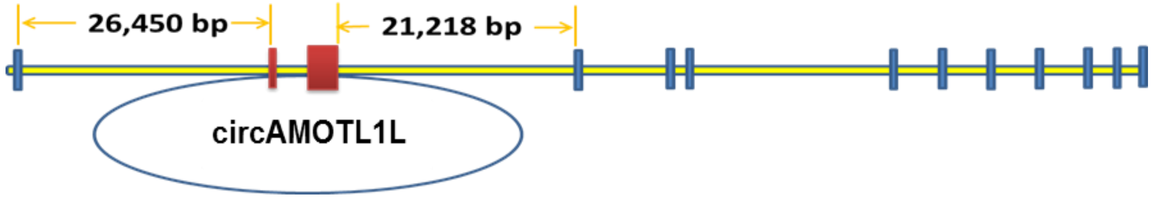

Supplementary Figure 2. Schematics showing that the exon-2 upstream intron (26,450 bp) and the exon-3 downstream intron (21,218 bp) in the *AMOTL1* gene contain, respectively, a long flanking sequence with complementary Alu repeats, which might facilitate the cyclization of a circRNA.

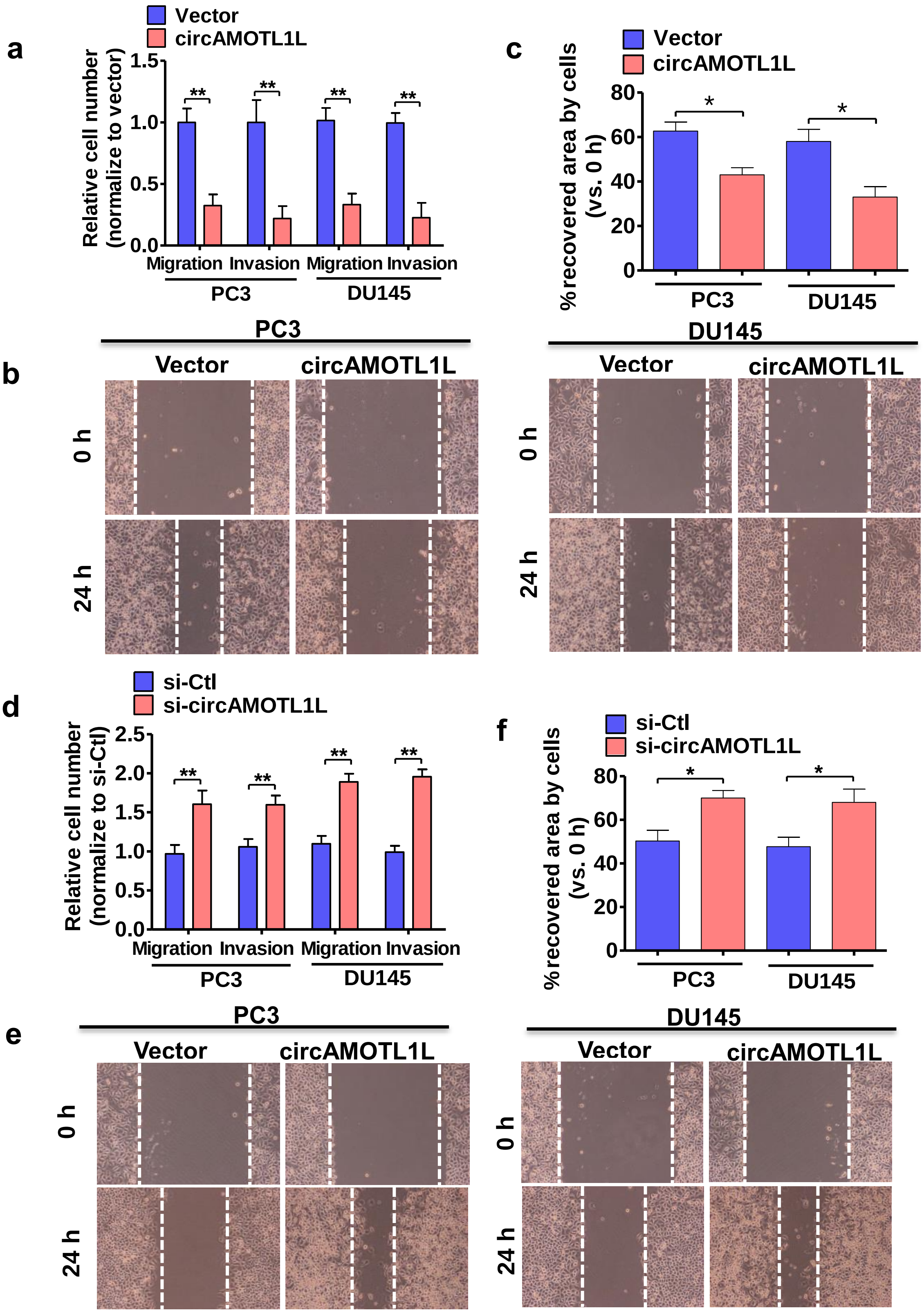

Supplementary Figure 3. Overexpression or knockdown of circAMOTL1L in PCa cell lines inhibits or promotes, respectively, cell migration in vitro. a**,** Quantitative analysis of Figure 2C. Data are expressed as mean±SEM from three independent experiments. ***P*<0.01 vs Vector. **b,** The migration ability of PC3 and DU145 cells transfected with circAMOTL1L or

empty vector was determined by wound healing assay. **c,** Quantitative analysis of (**b**). Bars are mean ± SEM of triplicate samples. **P*<0.05 vs. empty vector. **d,** Quantitative analysis of Figure 2E. Data are expressed as mean±SEM from three independent experiments. ***P*<0.01 vs Vector. **e,** The migration ability of PC3 and DU145 cells transfected with si-circAMOTL1L, si-linear AMOTL1 or si-Ctl was determined by wound healing assay. **f,** Quantitative analysis of (**e**). Bars are mean ± SEM of triplicate samples. **P*<0.05 vs. empty vector.

<table>
<tr><th>microRNA</th><th>position</th><th>matching</th></tr>
<tr><td>miR-193a-5p</td><td>6</td><td>CCTTCT-GCTTGTTATAGCCCCA
   ||| ||::|: || ||||
AGTAGAGCGGGCG-TTTCTGGGT</td></tr>
<tr><td>miR-193a-5p</td><td>377</td><td>AAACCTCACTCAAGAAGACCCA
    ||| |:| :||||||||
AGTAGAGCGGGCGTTTCTGGGT</td></tr>
<tr><td>miR-193a-5p</td><td>411</td><td>CAGTC-AGCACGCCAAGAACCG
  :|| || ||| |||| ||:
AGTAGAGCGGGCGTTTCTGGGT</td></tr>
<tr><td>miR-193a-5p</td><td>475</td><td>AGGTCCGGTCCACGCAGACTCA
  :|| |:|| |: ||||:||
AGTAGAGCGGGCGTTTCTGGGT</td></tr>
<tr><td>miR-193a-5p</td><td>857</td><td>AGTGCTGGACCCTCGGGGTCCTC
    || | ||| |:::||||:
AGTAGAGC-GGGCGTTTCTGGGT</td></tr>
<tr><td>miR-193a-5p</td><td>912</td><td>ATGTCCCCAGTCAGCAA-GACCCA
  :|| |:| |||| ||||||
AGTAG--AGCGGGCGTTTCTGGGT</td></tr>
</table>

Supplementary Figure 4. The prediction for miR-193a-5p binding sites on circAMOTL1L transcript. The red nucleotides are the seed sequences of miR-193a-5p.

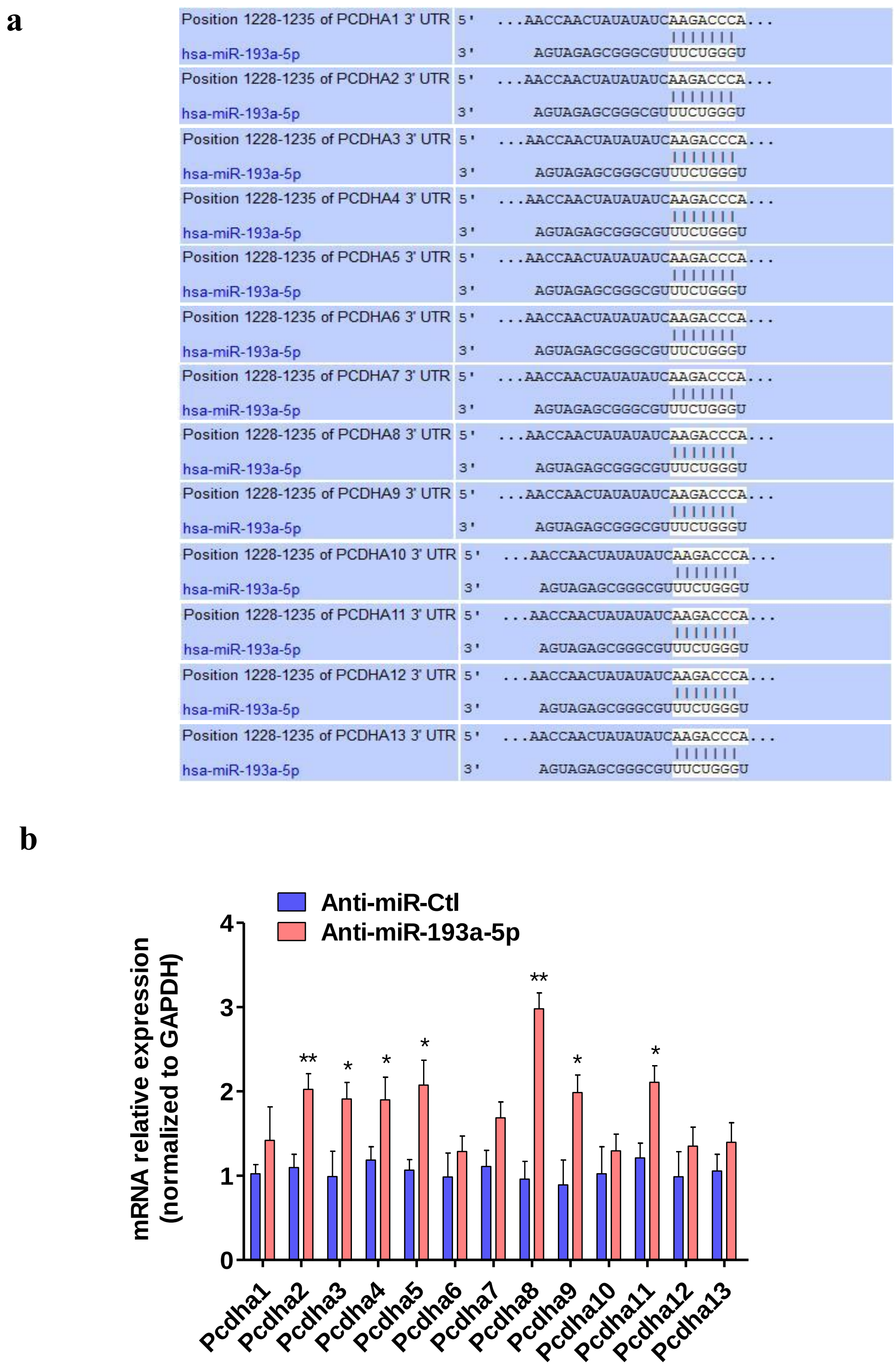

Supplementary Figure 5. a, miR-193a-5p targets the mRNA 3' UTR of Pcdha gene cluster as predicted by using Targetscan (http://www.targetscan.org/vert_71). **b,** PC3 cells were transfected with anti-miR-193a-5p or anti-miR-Ctl for 24 h, and then qRT-PCR detected the mRNA expression of Pcdha gene cluster with the primers indicated in Supplementary Table 2. *$P < 0.05$, **$P < 0.01$ vs. GAPDH.

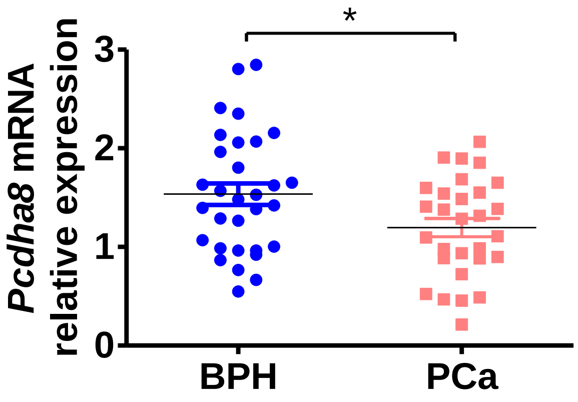

Supplementary Figure 6. Quantitative real-time (qRT)-PCR analysis of *Pcdha8* mRNA expression in benign prostatic hyperplasia (BPH, n=31) and PCa tissues (PCa, n=29). *p<0.05 vs. BPH.

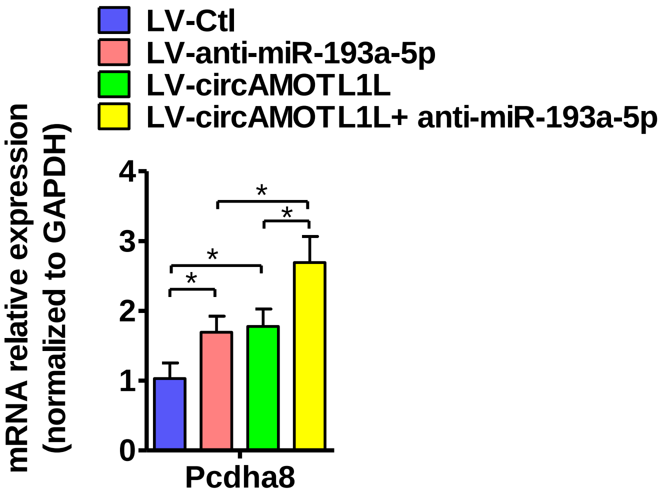

Supplementary Figure 7. Quantitative real-time (qRT)-PCR analysis of Pcdha8 mRNA expression in the xenograft tumor tissues. *p<0.05.

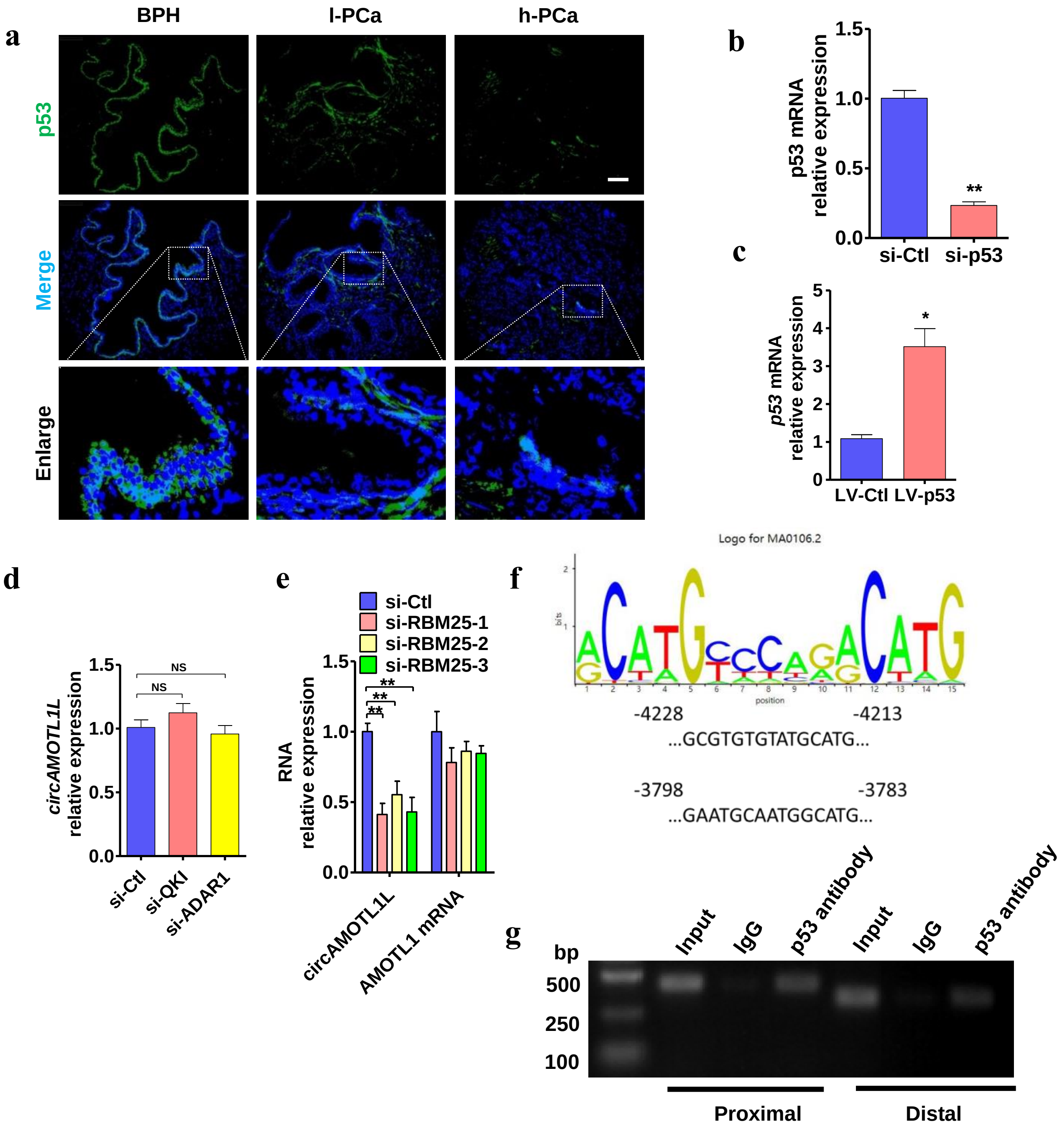

Supplementary Figure 8. a, Immunostaining detected p53 expression in BPH, low grade PCa and high grade PCa tissues. The expression of p53 was downregulated in the two PCa tissues compared to the BPH tissues. Scale bar =32 µm. **b,** PC3 cells were transfected with p53-specific siRNA (si-p53) or control siRNA (si-Ctl), and then qRT-PCR examined the expression of p53 mRNA. $^{**}P < 0.01$ vs. si-Ctl. **c,** qRT-PCR detected p53 mRNA in PC3 cells stably transfected with lentivirus (LV)-p53 or LV-Ctl constructs. $^{*}P < 0.05$ vs. LV-Ctl. **d,** qRT-PCR detected the expression of circAMOTL1L in PC3 cells transfected with siRNAs *specific for QKI or ADAR1*. **e,** PC3 cells were transfected with three different siRNAs *specific for* RBM25 or control siRNA, and then qRT-PCR detected circAMOTL1L and AMOTL1 mRNA expression. $^{**}P < 0.01$ vs. si-Ctl. **f,** There are two putative p53 binding elements within the 5 Kb promoter region of RBM25. **g,** ChIP-qPCR detected p53 binding to the different regions of the RBM25 promoter in PC3 cells.

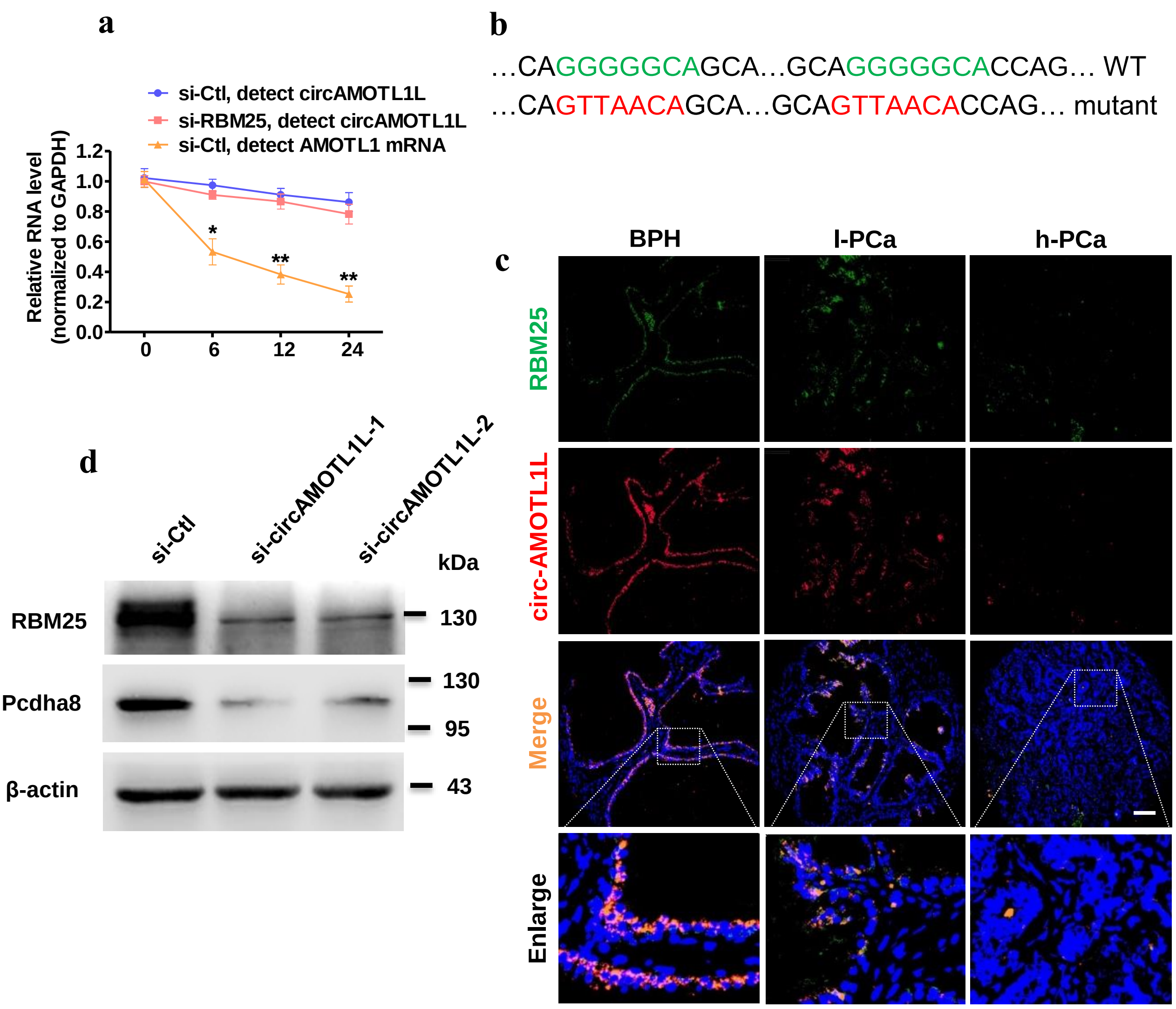

Supplementary Figure 9. a, PC3 cells were transfected with RBM25-specific siRNA (si-RBM25) or si-Ctl, and then treated with actinomycin D. qRT-PCR detected the level of circAMOTL1L and AMOTL1 mRNA. *P<0.05, **P<0.01 vs. 0 h. **b,** The RBM25 binding sites in circAMOTL1L sequence. WT sequence were marked in green and mutant sequence in red. **c,** RBM25 expression and its co-localization with circAMOTL1L were detected by immunostaining with the anti-RBM25 combined with *in situ* hybridization of circAMOTL1L in BPH, low grade PCa and high grade PCa tissues. Scale bar = 32 μm. **d,** PC3 cells were transfected with two different siRNAs specific for circAMOTL1L or control siRNA (si-Ctl), and then Western blotting detected RBM25 and Pcdha8 expression.
