# Supplementary material for: Dysregulation of p53-RBM25-mediated circAMOTL1L biogenesis contributes to prostate cancer progression through the circAMOTL1L-miR-193a-5p-Pcdha pathway
Source: Oncogene. 2018 Dec 7;38(14):2516–32. doi: 10.1038/s41388-018-0602-8 (PMC6484770; doi:10.1038/s41388-018-0602-8)
Supplement: Supplementary file 4 — Supplementary Table 2 [file 41388_2018_602_MOESM4_ESM.docx]

**Supplementary table 2** Oligos used in the study

| **Oligo name** | **Sequence (5' - 3')** | **Purpose** | **Amplicon (bp)** |
| --- | --- | --- | --- |
| circAMOTL1L-F: | CCACGAGATGGTCAAGCCCTAC | Real-time PCR and qRT-PCR for circAMOTL1L. | 216 |
| circAMOTL1L-R: | CCACCGTCAAAGCAGATGTTTC |  |  |
| linerAMOTL1L-F: | GGAATGGGGCCAAGCAACAC | Real-time PCR and RT-PCR for AMOTL1L mRNA. | 195 |
| linerAMOTL1L-F: | GGTCACCATAAAAAAGTCCGTGC |  |  |
| circCHSY1-F: | CTACTGGAATTGGTACAGATGTGTCAGAACC | RT-PCR for circCHSY1. | 130 |
| circCHSY1-R: | GAAGGTGTGTCCGGAGGTTTGCAG |  |  |
| linerCHSY1-F: | CTCCCGGAGACACTTGCCAATGTG | RT-PCR for linerCHSY1. | 146 |
| linerCHSY1-R: | GGCAGACAGGCCTGGGCACC |  |  |
| circFAM190B-F: | CCCAAACACCCTGGAGACGAA | RT-PCR for circFAM190B. | 174 |
| circFAM190B-R: | GCAATCCTGAACCATGTGCCG |  |  |
| linerFAM190B-F: | CTCCCTATAGAGAATCTCCTTTGGGTC | RT-PCR for linerFAM190B. | 143 |
| linerFAM190B-R: | GCAATCCTGAACCATGTGCCG |  |  |
| circROCK2-F: | GCTTGTGAAGGTAGCACGATTTCTAA | RT-PCR for circROCK2 | 111 |
| circROCK2-R: | GATGTTGTAAAAGTTATTGGAAGAGGTGC |  |  |
| linerROCK2-F: | GGACTGCCCCTATCATGTTTTCCTG | RT-PCR for linerROCK2 | 183 |
| linerROCK2-R: | GCAGGTTTAATGAGAGGGAACTTAGTGTAGC |  |  |
| GAPDH-F: | AAGGTGAAGGTCGGAGTCAAC | Real-time PCR and qRT-PCR for GAPDH | 102 |
| GAPDH-R: | GGGGTCATTGATGGCAACAATA |  |  |
| pcDNA-AMOTL1-inf-F: | CATTCCCTTTCTTTCCCTCAGGGTCCCCTTCTGCTTGTTATAGCCC | Clone the overexpression vector for circAMOTL1L | 1072 |
| pcDNA-AMOTL1-inf-R: | CGTCTGCAGTTGATACTCACCTCGTTACCCCATACTCAGGGGGTG |  |  |
| FL-circAMOTL1L-F: | CCTGAGTATGGGGTAACGAGGGTCCC | Identify the full-long circAMOTL1L | 1072 |
| FL-circAMOTL1L-R: | CAAGCAGAAGGGGACCCTCGTTACC |  |  |
| circAMOTL1L-T7-F: | GCCTGCAGGTAAAGTGCTGGACCC | cricAMOTL1L probe primer for northern blot | 422 |
| circAMOTL1L-T7-R: | GGATCCTAATACGACTCACTATAGGCCTCAGGAAGTTTGGGGAGTGGAAG |  |  |
| Linear-AMOTL1L- T7-F: | CATCAACCATGCAGCAGCACAGC | AMOTL1 mRNA probe primer for northern blot | 603 |
| Linear-AMOTL1L- T7-R: | GGATCCTAATACGACTCACTATAGGGGTCCAGGATCTCGATGTGTCTCCG |  |  |
| Biotin-con-1: | CTTCACCCCAGGAACAAACTCCTTTGCATT | Oligo-pulldown NC Biotin | N/A |
| Biotin-con-2: | CAGCTGGATTATGGGTTGGTATTGGTCATA |  |  |
| Biotin-con-3: | GGCTGGACTGGCACTCATCTGCTGACTAAC |  |  |
| circAMOTL1L-biotin-1: | CTATAACAAGCAGAAGGGGACCCTCGTTAC | Oligo-pulldown | N/A |
| circAMOTL1L-biotin-2: | CAAGCAGAAGGGGACCCTCGTTACCCCATAC |  |  |
| circAMOTL1L-biotin-3: | GCAGAAGGGGACCCTCGTTACCCCATACTC |  |  |
| GLO-circAMOTL1L-1072-F: | GCTCGCTAGCCTCGAGGGTCCCCTTCTGCTTGTTATAGCC | Pmir-GLO-circAMOTL1L vector for Dual-Glo Luciferase Assay | 1072 |
| GLO-circAMOTL1L-1072-R: | ATGCCTGCAGGTCGACCTCGTTACCCCATACTCAGGGG |  |  |
| circAMOTL1L-FAM: | GAGTATGGGGTAACGAGGGTCCCCTTCTGC | FISH | N/A |
| circAMOTL1L-cy3: | GAGTATGGGGTAACGAGGGTCCCCTTCTGC | FISH | N/A |
|  |  |  |  |
| has-vimentin-F: | GGACCAGCTAACCAACGACA | Real-time PCR for vimentin mRNA. | 178 |
| has-vimentin-R: | AAGGTCAAGACGTGCCAGAG |  |  |
| has-Ecadherin-F: | GCTGGACCGAGAGAGTTTCC | Real-time PCR for E-cadherin mRNA. | 155 |
| has-Ecadherin-R: | CAAAATCCAAGCCCGTGGTG |  |  |
| has-β-catenin-F: | GAGCCTGTTCCCCTGAGGGTATTTG | Real-time PCR for β-catenin mRNA. | 416 |
| has-β-catenin-R: | GGACTGAGAAAATCCCTGTTCCCAC |  |  |
| has-Ncadherin-F: | GGGAAATGGAAACTTGATGGC | Real-time PCR for Ncadherin mRNA | 205 |
| has-Ncadherin-R: | AGCTTCTCACGGCATACACC |  |  |
| has-ZO-1-F: | GCAAGAGATGGCAATATTCAAGAAGGTG | Real-time PCR for ZO-1 mRNA. | 343 |
| has-ZO-1-R: | GCTGCGGCGAGTGCCTGGAATG |  |  |
| miR-193a-5p-F | CTGGGTCTTTGCGGGCGAG | Real-time PCR for miR-193a-5p | N/A |
| miR-193b-5p-F | GTCGGGGTTTTGAGGGCGAG | Real-time PCR for miR-193b-5p | N/A |
| miR-23b-5p-F | CGTGGGTTCCTGGCATGCTG | Real-time PCR for miR-23b-5p | N/A |
| miR-339-5p-F | GCCTCCCTGTCCTCCAGGAGC | Real-time PCR for miR-339-5p | N/A |
| miR-125a-5p-F | CGTCCCTGAGACCCTTTAACCTGTG | Real-time PCR for miR-125a-5p | N/A |
| miR-92a-2-5p-F | CGGGGGTGGGGATTTGTTGC | Real-time PCR for miR-92a-2-5p | N/A |
| RNU6-1(U6)-F | GTGCTCGCTTCGGCAGCACATATAC | Real-time PCR for the internal control U6 | 106 |
| RNU6-1(U6)-R | AAAATATGGAACGCTTCACGAATTTGC |  |  |
| miR-129-5p | CTTTTTGCGGTCTGGGCTTG | Real-time PCR for miR-129-5p | N/A |
| miR-149-3p | AGGGAGGGACGGGGGCTG | Real-time PCR for miR-149-3p | N/A |
| miR-191-3p | GCTGCGCTTGGATTTCGTCC | Real-time PCR for miR-191-3p | N/A |
| miR-328-5p | GGGGGGGCAGGAGGGGCTC | Real-time PCR for miR-328-5p | N/A |
| miR-345-3p | GCCCTGAACGAGGGGTCTGG | Real-time PCR for miR-345-3p | N/A |
| miR-370-3p | GCCTGCTGGGGTGGAACCTG | Real-time PCR for miR-370-3p | N/A |
| miR-375 | GTTTGTTCGTTCGGCTCGCG | Real-time PCR for miR-375 | N/A |
| miR-378a-5p | GGCTCCTGACTCCAGGTCCTGTG | Real-time PCR for miR-378a-5p | N/A |
| miR-513a-5p | GGCCTTCACAGGGAGGTGTCAT | Real-time PCR for miR-513a-5p | N/A |
| miR-453 | AGGTTGTCCGTGGTGAGTTCG | Real-time PCR for miR-453 | N/A |
| miR-608 | AGGGGTGGTGTTGGGACAGCTC | Real-time PCR for miR-608 | N/A |
| miR-612 | GCTGGGCAGGGCTTCTGAGC | Real-time PCR for miR-612 | N/A |
| miR-670-5p | GGCGTCCCTGAGTGTATGTGGTG | Real-time PCR for miR-670-5p | N/A |
| miR-762 | GGGGCTGGGGCCGGGGCC | Real-time PCR for miR-762 | N/A |
| miR-943 | CTGACTGTTGCCGTCCTCCAG | Real-time PCR for miR-943 | N/A |
| miR-1229-5p | GTGGGTAGGGTTTGGGGGAGAG | Real-time PCR for miR-1229-5p | N/A |
| miR-1238-5p | GTGAGTGGGAGCCCCAGTGTG | Real-time PCR for miR-1238-5p | N/A |
| miR-1251-3p | GCGCTTTGCTCAGCCAGTGTAG | Real-time PCR for miR-1251-3p | N/A |
| miR-193a-5p-FAM: | CCCAGAAACGCCCGCTCTAC | FISH | N/A |
| miR-193a-5p-biotin: | AGTAGAGCGGGCGTTTCTGGGT | Oligo-pulldown | N/A |
| miR-193b-5p-biotin | CGGGGTTTTGAGGGCGAGATGA | Oligo-pulldown | N/A |
| miR-339-5p-biotin | TCCCTGTCCTCCAGGAGCTCACG | Oligo-pulldown | N/A |
| PCDHA1-F: | CACCGCCTGAGAGAAGACAG | Real-time PCR for PCDHA family genes | 218 |
| PCDHA1-R: | GATCGAGTAGTGGAGCTGGC |  |  |
| PCDHA2-F: | TCCAGACTCCGCAGAAGAGA |  | 94 |
| PCDHA2-R: | GTTTCAAGAGGCAAGCTGGA |  |  |
| PCDHA3-F: | TGTCCAGTCTGTTGGTGCTC |  | 345 |
| PCDHA3-R: | CCAGAGGAGTGGAAACATTGAG |  |  |
| PCDHA4-F: | TGATCAGCGTGTCCGACAAA |  | 296 |
| PCDHA4-R: | CGGGTTGTTCTCCTTCACGA |  |  |
| PCDHA5-F: | GTGAAACTCCTGGATGTGAATGATAATACC |  | 177 |
| PCDHA5-R: | CTTGAAGGGAACGTGGGGCATTAG |  |  |
| PCDHA6-F: | CTGTTGAATGATGGCGGACG |  | 162 |
| PCDHA6-R: | GGGAGGAGCAGACATTGCTT |  |  |
| PCDHA7-F: | CCGAATGGATACGACCCAGG |  | 118 |
| PCDHA7-R: | TGCCATGTTTAGCCTCCTCG |  |  |
| PCDHA8-F: | CCAGAATGCCAGACTCTCGGTTTC |  | 220 |
| PCDHA8-R: | CCCCCATCTGTGGCTGTCAGG |  |  |
| PCDHA9-F: | GATGTCACTCTTTGCCGCGATGTTC |  | 137 |
| PCDHA9-R: | GATTTCGTCAGTCCCATTCTGCTG |  |  |
| PCDHA10-F: | AGGCATCAGCCAGTTTCTCAA |  | 378 |
| PCDHA10-R: | AAGGTCCCCGTGTCTTTTGG |  |  |
| PCDHA11-F: | GCCCCAGTCTTCCTCTAGGT |  | 115 |
| PCDHA11-R: | GAGGCAGAGTAACGCCAGTC |  |  |
| PCDHA12-F: | GCGCCTCTGGACTCTCATTT |  | 383 |
| PCDHA12-R: | AGTCCTTCGTCTGGATCGGA |  |  |
| PCDHA13-F: | AGAGGGAGGAGGACTCAGAAT |  | 135 |
| PCDHA13-R: | CTGGACCAGCCCGTAGAATG |  |  |
| QKI-F: | GCCTGATGCTGTGGGACCTATTG | Real-time PCR for QKI mRNA | 210 |
| QKI-R: | GGCTTGCCTCTATTTTGCTCCTCC |  |  |
| ADAR1-F: | GCGACTATCTCTTCAATGTGTCTGACTC | Real-time PCR for ADAR1 mRNA | 289 |
| ADAR1-R: | GAGGCATTTGATGTGGGTATATTACAGG |  |  |
| p53-F: | CCAGATGAAGCTCCCAGAATGCC | Real-time PCR for TP53 mRNA | 178 |
| p53-R: | CTGTCCCAGAATGCAAGAAGCCC |  |  |
| NONO-F: | GCCTCGGGAGCTAAACCTTG | Real-time PCR for NONO mRNA | 253 |
| NONO-R: | CGCCTAAATACTGGGCCTCTC |  |  |
| FXR1-F: | GGGTGGTGGTTGGCTAAAGT | Real-time PCR for FXR1 mRNA | 113 |
| FXR1-R: | ACAGGCCGAAGTCGTTCAAA |  |  |
| RBM25-F: | CGTGCACTCAGATTATTACATGACCTGC | Real-time PCR for RBM25 mRNA | 250 |
| RBM25-R: | GGGCATTTAGCTCACTGGAGTATTCACG |  |  |
| PUM1-F: | GGGATAACATTCATGCAGAACATCAGG | Real-time PCR for PUM1 mRNA | 213 |
| PUM1-R: | CTGAATGATCTGATGTTCCCCAGGC |  |  |
| ZNF385A-F: | GCGGCTGCTCTACTGTGCTCTGTG | Real-time PCR for ZNF385A mRNA | 217 |
| ZNF385A-R: | GACCTTGACATTGCAGATCTCACAGTGG |  |  |
| RBM25-pro-chip-F1: | GCCATTATATACTATATAATGGCATTG | CHIP PCR for detect p53 binding region. | 242 |
| RBM25-pro-chip-R1: | GCTGGAACTGAGCAGATCTTCTC |  |  |
| RBM25-pro-chip-F2: | GGCTAACATGGAGAAACCCCACC |  | 250 |
| RBM25-pro-chip-R2: | GATATTACATTCTGTGTGCATTTGGACAC |  |  |
| RBM25-pro-chip-F3: | GGGTGGCCTCGAACTCCTAACC |  | 317 |
| RBM25-pro-chip-R3: | GAAACATACTACACACTTCCTGCCTGC |  |  |
| RBM25-Prom-inf-F: | CCGAGCTCTTACGCGTCTTCAGTTACTCCACCTTGCTGGGTAG | Clone RBM25 promoter. | 1799 |
| RBM25-Prom-inf-R: | GATCGCAGATCTCGAGTCGTCTCCGCTGTTGGCTCTG |  |  |
|  |  |  |  |
| si-circAMOTL1L-1-F | CGAGGGUCCCCUUCUGCUUTT | For knock-down circAMOTL1L. | N/A |
| si-circAMOTL1L-1-R | AAGCAGAAGGGGACCCUCGTT |  |  |
| si-circAMOTL1L-2-F | GGGUAACGAGGGUCCCCUUTT | For knock-down circAMOTL1L. | N/A |
| si-circAMOTL1L-2-R | AAGGGGACCCUCGUUACCCTT |  |  |
| si-linearAMOTL1L-1-F | GCCCAUCCUACAAACAACUTT | For knock-down both circAMOTL1L and AMOTL1L mRNA. | N/A |
| si-linearAMOTL1L-1-R | AGUUGUUUGUAGGAUGGGCTT |  |  |
| si-linearAMOTL1L-2-F | CCCUUCAAGACCAAGCAAATT | For knock-down both circAMOTL1L and AMOTL1L mRNA. | N/A |
| si-linearAMOTL1L-2-R | UUUGCUUGGUCUUGAAGGGTT |  |  |
| si-PCDHA8-1-F: | GGAGCUAACUCCGUGUUAATT | For knock-down PCDHA8 mRNA. | N/A |
| si-PCDHA8-1-R: | UUAACACGGAGUUAGCUCCTT |  |  |
| si-PCDHA8-2-F: | GCUCUCAUGAUUACUUCAUTT | For knock-down PCDHA8 mRNA. | N/A |
| si-PCDHA8-2-R: | AUGAAGUAAUCAUGAGAGCTT |  |  |
| si-NC-F | UUCUCCGAACGUGUCACGUTT | si-RNA control. | N/A |
| si-NC-R | ACGUGACACGUUCGGAGAATT |  |  |
| si-RBM25-1-F: | GGAGCUCAGAUCGUAAUAATT | For knock-down RBM25 | N/A |
| si-RBM25-1-R: | UUAUUACGAUCUGAGCUCCTT |  |  |
| si-RBM25-2-F: | CAGACAUGCUUAUAAGACATT |  |  |
| si-RBM25-2-R: | UGUCUUAUAAGCAUGUCUGTT |  |  |
| si-RBM25-3-F: | CUGGAAGAGAGUACAAGGUTT |  |  |
| si-RBM25-3-R: | ACCUUGUACUCUCUUCCAGTT |  |  |
| si-p53 | sc-29435, santa cruz | For knock-down TP53 | N/A |
| si-ADAR1-F | CGCAGAGUUCCUCACCUGUTT | For knock-down ADAR1 | N/A |
| si-ADAR1-R | ACAGGUGAGGAACUCUGCGTT |  |  |
| si-QKI-F | GCUGCUCCAAGGAUCAUUATT | For knock-down QKI | N/A |
| si-QKI-R | UAAUGAUCCUUGGAGCAGCTT |  |  |
| si-FXR1-F | GGGAUCCCUAACGUAAUAUTT | For knock-down FXR1 | N/A |
| si-FXR1-R | AUAUUACGUUAGGGAUCCCTT |  |  |
| si-NAF1-F | CAGCUGGAAACUCUGAAAUTT | For knock-down NAF1 | N/A |
| si-NAF1-R | AUUUCAGAGUUUCCAGCUGTT |  |  |
| si-NONO-F | GAGGUGCUAUGGGCAUAAATT | For knock-down NONO | N/A |
| si-NONO-R | UUUAUGCCCAUAGCACCUCTT |  |  |
| si-RNPS1-F | GAGCUUGCUAGGAGUCAAATT | For knock-down RNPS1 | N/A |
| si-RNPS1-R | UUUGACUCCUAGCAAGCUCTT |  |  |
| si-SART3-F | GCCAGAAGAUGAGUGAAAUTT | For knock-down SART3 | N/A |
| si-SART3-R | AUUUCACUCAUCUUCUGGCTT |  |  |
| si-TRA2A-F | CGGGUGGAUUAUUCUAUAATT | For knock-down TRA2A | N/A |
| si-TRA2A-R | UUAUAGAAUAAUCCACCCGTT |  |  |
| si-CIRBP-F | GAGCAGGUCUUCUCAAAGUTT | For knock-down CIRBP | N/A |
| si-CIRBP-R | ACUUUGAGAAGACCUGCUCTT |  |  |
| si-EIF3G-F | GGAGGAAGAACUGGAAGAAUU | For knock-down EIF3G | N/A |
| si-EIF3G-R | UUCUUCCAGUUCUUCCUCCTT |  |  |
| si-PTBP1-F | CUGCAGCAAACGGAAAUGATT | For knock-down PTBP1 | N/A |
| si-PTBP1-R | UCAUUUCCGUUUGCUGCAGTT |  |  |
| si-RBM28-F | GCCGUGGCAAAGGAUAAAUTT | For knock-down RBM28 | N/A |
| si-RBM28-R | AUUUAUCCUUUGCCACGGCTT |  |  |
| si-RBM7-F | GGGCAACCUUGAAACGAAATT | For knock-down RBM7 | N/A |
| si-RBM7-R | UUUCGUUUCAAGGUUGCCCTT |  |  |
| si-SLIRP-F | GCUGCGUAGAAGUAUCAAUTT | For knock-down SLIRP | N/A |
| si-SLIRP-R | AUUGAUACUUCUACGCAGCTT |  |  |
| si-SRP14-F | GCUCCAAGGAAGUGAAUAATT | For knock-down SRP14 | N/A |
| si-SRP14-R | UUAUUCACUUCCUUGGAGCTT |  |  |
| si-SUGP1-F | GCAGGGAAUUCCUCUACUATT | For knock-down SUGP1 | N/A |
| si-SUGP1-R | UAGUAGAGGAAUUCCCUGCTT |  |  |
| p53-sgRNAs-1: | TTGCCGTCCCAAGCAATGGA | Knockout *TP53* | N/A |
| p53-sgRNAs-2: | CCCCGGACGATATTGAACAA |  |  |
| RBM25-binding-sgRNAs-1: | CAAAGCACAGTCGCAGTTCTT | Knockout RBM25 binding site of circAMOTL1. | N/A |
| RBM25-binding-sgRNAs-2: | CCACTGTGAACCGTGCCAACA |  |  |
| 5’ Alu sequences | CGGGCGCGGTGCCTCACGCCTGTAATCCCAGCACTTTGGGAGCCTGGGGC | For constructs a circ-pcDNA3.1 vector. | N/A |
| 3’ Alu sequences | GCCCCAGGCTCCCAAAGTGCTGGGATTACAGGCGTGAGGCACCGCGCCCG |  |  |
| 5' donor splice sequences | ACTCTGACAATTCCCTTTCTTTCCCTCAG |  |  |
| 3' acceptor splice sequences | GTGAGTATCAACTGCAGACGTTTCGTGCGGC |  |  |
