# Supplementary material for: Dysregulation of p53-RBM25-mediated circAMOTL1L biogenesis contributes to prostate cancer progression through the circAMOTL1L-miR-193a-5p-Pcdha pathway
Source: Oncogene. 2018 Dec 7;38(14):2516–32. doi: 10.1038/s41388-018-0602-8 (PMC6484770; doi:10.1038/s41388-018-0602-8)
Supplement: Supplementary file 6 — Supplementary Table 4 [file 41388_2018_602_MOESM6_ESM.docx]

| **Gene names** | **Protein names** |
| --- | --- |
| ACTN1 | Alpha-actinin-1 |
| KRT14 | cytoskeletal 14 |
| PTMA | Prothymosin alpha |
| HSPD1 | 60 kDa heat shock protein |
| CFL1 | Cofilin-1 |
| RPL18 | 60S ribosomal protein L18 |
| HNRNPL | Heterogeneous nuclear ribonucleoprotein L |
| ATP5A1 | ATP synthase subunit alpha |
| CCT7 | T-complex protein 1 subunit |
| LAMP2 | Lysosome-associated membrane glycoprotein 2 |
| RPL23A | 60S ribosomal protein L23a |
| NCL | Nucleolin |
| SSBP1 | Single-stranded DNA-binding protein |
| KRT5 | cytoskeletal 5 |
| H2AFV | Histone H2A.V |
| SF3A3 | Splicing factor 3A subunit 3 |
| KRT16 | cytoskeletal 16 |
| CALR | Calreticulin |
| NONO | Non-POU domain-containing octamer-binding protein |
| CLTC | Clathrin heavy chain |
| HEL70 | Moesin |
| KRT9 | cytoskeletal 9 |
| TXN | Thioredoxin |
| RBM25 | RNA-binding protein 25 |
| DCTPP1 | dCTP pyrophosphatase 1 |

Supplementary Table 4 Co-precipitating proteins with circAMOTL1L
